# A multivariate study of differentiating characters between three European species of the genus *Lasiochernes* Beier, 1932 (Pseudoscorpiones, Chernetidae)

**DOI:** 10.3897/zookeys.629.8445

**Published:** 2016-11-07

**Authors:** Jana Christophoryová, Katarína Krajčovičová, Hans Henderickx, Stanislav Španiel

**Affiliations:** 1 Department of Zoology, Faculty of Natural Sciences, Comenius University, Mlynská dolina, Ilkovičova 6, SK–842 15 Bratislava, Slovakia; 2 Department of Biology, Universiteit Antwerpen (UA), Groenenborgerlaan 171, 2020 Antwerpen, Belgium; 3 Royal Belgian Institute of Natural Sciences, Department of Entomology, Vautierstraat 29, B–1000, Brussels; 4 Institute of Botany, Slovak Academy of Sciences, Dúbravská cesta 9, SK–845 23 Bratislava, Slovakia; 5 Department of Botany, Faculty of Science, Charles University, Benátská 2, CZ–128 01 Praha, Czech Republic

**Keywords:** Caves, mole nests, morphology, morphometric analysis, pseudoscorpion, taxonomy

## Abstract

Morphological variation in three rarely collected European species of the genus *Lasiochernes* Beier, 1932 is thoroughly examined in the present study. Detailed descriptions of previously ignored morphological characters of *Lasiochernes cretonatus* Henderickx, 1998, *Lasiochernes jonicus* (Beier, 1929) and *Lasiochernes pilosus* (Ellingsen, 1910) are presented. The female of *Lasiochernes cretonatus* and the nymphs of *Lasiochernes pilosus* are described for the first time. Multivariate morphometric techniques (principal coordinate analysis and discriminant analyses) were employed to confirm morphological differentiation of the three *Lasiochernes* species and to identify the most reliable characters for their separation. The usefulness of particular body parts for species identification was evaluated. An identification key for the females of the *Lasiochernes* species studied is provided. Geographic distribution and habitat preferences of the three species are summarized.

## Introduction

The genus *Lasiochernes* Beier, 1932 belongs to the subfamily Lamprochernetinae, as defined by Harvey (1994). Until now, ten species of the genus have been discovered (Harvey 2013). They are rarely collected, usually being found in the nests of small mammals or in caves. The genus is characterized by the presence of a long tactile seta on pedal tarsus IV, a pair of long tactile setae on tergite XI, five setae on the hand of the chelicera, secondary sexual dimorphism of the setation of the palps, with male palps bearing a long, dense setation, and a T-shaped spermatheca in females. Most of the known species are recorded from only one or two countries: *Lasiochernes anatolicus* Beier, 1963 and *Lasiochernes villosus* Beier, 1957 from Turkey; *Lasiochernes turcicus* Beier, 1949 from Turkey and Israel; *Lasiochernes congicus* Beier, 1959 and *Lasiochernes punctiger* Beier, 1959 from the Democratic Republic of Congo; *Lasiochernes jonicus* (Beier, 1929) and *Lasiochernes cretonatus* Henderickx, 1998 from Greece; *Lasiochernes graecus* Beier, 1963 from Albania and Greece and *Lasiochernes siculus* from Italy (Harvey 2013). Only *Lasiochernes pilosus* (Ellingsen, 1910) occurs in several European countries (Harvey 2013).

Detailed morphological descriptions of European pseudoscorpion species are rare. This holds true for both the adults and nymphal stages. These descriptions of adults and all nymphal stages are available mainly for the families Chthoniidae, Neobisiidae and Cheliferidae (e.g. Gabbutt and Vachon 1963, 1965, 1967, 1968, Gabbutt 1970), rarely for the family Chernetidae (Sezek and Özkan 2007, Christophoryová et al. 2012).

Material of three *Lasiochernes* species was obtained during our study: *Lasiochernes cretonatus*, *Lasiochernes jonicus* and *Lasiochernes pilosus*. *Lasiochernes cretonatus* was described from a single male collected in a cave in Crete (Greece) (Henderickx 1998). *Lasiochernes jonicus* was briefly described by Beier (1929), based on several adult specimens from Corfu, Greece. *Lasiochernes pilosus* is distributed in several European countries (Harvey 2013) and it shows a degree of host-specificity, since it is almost exclusively found in subterranean mole-nests with a particular content of dead leaves. Many adults and nymphal stages of the latter species had been collected, but there had been no detailed description of nymphs and some characters of the adults remained unknown.

Morphological differences between species of pseudoscorpions, as reported in taxonomic descriptions, are often based on quantitative traits. Multivariate morphometric methods are an effective tool to compare the role of numerous quantitative and qualitative characters and allow in-depth examination of morphological variation of phenetically similar taxa. In recent years, many papers have successfully employed multivariate morphometrics in the taxonomy of invertebrates, such as mites (Klimov et al. 2004, Stekolnikov et al. 2010, Jagersbacher-Baumann 2014), flies (Castañeda et al. 2015, Van Cann et al. 2015), beetles (Sha et al. 2016) and spiders (Hamilton et al. 2016). The applicability of these methods for differentiation of pseudoscorpion species has been studied on the family of Chthoniidae. Muster et al. (2004) used multivariate analyses to separate two European species of the genus *Chthonius*.

The aims of this study are to (1) assemble detailed morphological descriptions of the adults of the three investigated *Lasiochernes* species, (2) describe all the nymphal stages of *Lasiochernes pilosus*, (3) assess the extent of morphological differentiation between adults of the three species, (4) identify the morphological characters that are most relevant for the differentiation of the three species and (5) provide an identification key for the females of the three species.

## Material and methods

**Figure 1.**
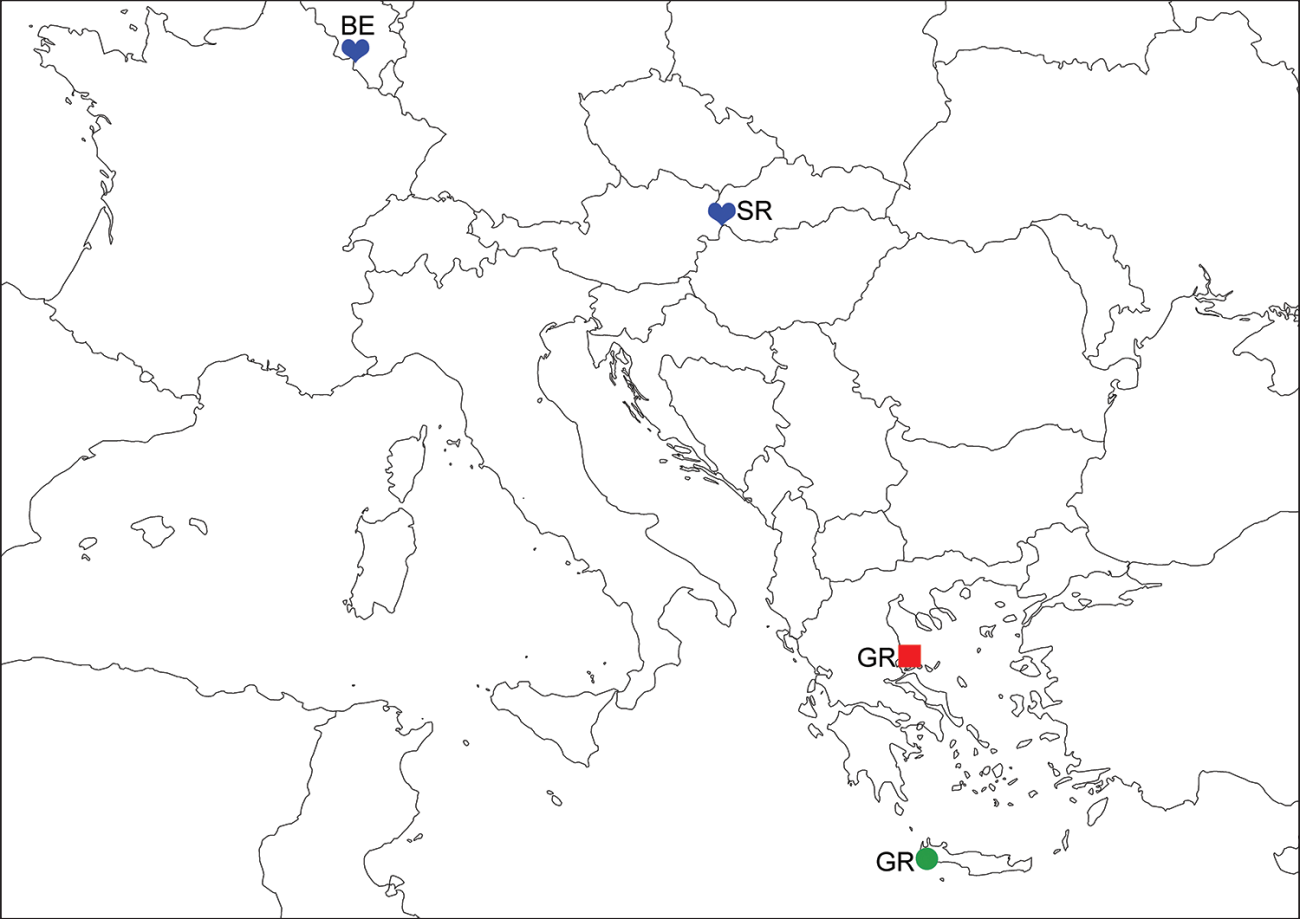
Collection localities of the studied material: *Lasiochernes cretonatus* (green circle), *Lasiochernes jonicus* (red square) and *Lasiochernes pilosus* (blue hearts).

*Lasiochernes cretonatus*: Greece, Crete, Azogires (Fig. 1), collected in Cave of 99 Holy Fathers/Souré Cave (35°16'22"N, 23°42'39"E; 500 m a.s.l.), 8 October 2000, one male, four females, leg. H. Henderickx.

*Lasiochernes jonicus*: Greece, Pelion, Mouresi (Fig. 1), collected in Tsouka cave (39°23'52"N, 23°10'12"E; 200 m a.s.l.), 3 November 2012, one male, one female, leg. H. Henderickx.

*Lasiochernes pilosus*: Slovakia, Malé Karpaty Mts., Borinka (Fig. 1), collected in nest of mole *Talpa europaea* Linnaeus, 1758 (48°15'44"N, 17°05'10"E; 300 m a.s.l.), 20 January 1990, three males, four females, 15 tritonymphs, 15 deutonymphs, 15 protonymphs, leg. Oto Majzlan. Belgium, Namur, Hastière (Fig. 1), collected in a *Talpa europaea* nest (50°13'10"N, 04°50'12"E; 200 m a.s.l.), 11 May 2001, two males, three females, leg. H. Henderickx.

Populations of *Lasiochernes* collected from mole nests in Belgium and Slovakia were identified as *Lasiochernes pilosus* (Beier 1963, Christophoryová et al. 2011) based on the setation on male palps and the habitat preference of this species. The taxonomic assignment of these two populations to *Lasiochernes pilosus* is also in agreement with the known geographic distribution of this species (Harvey 2013). The studied population of *Lasiochernes* from Crete is from the type locality of *Lasiochernes cretonatus*, a single cave at Azogires. The identification of this population as *Lasiochernes cretonatus* is supported by morphological characters mentioned in the original description of this species, namely the setation of the male palp and the position of the tactile seta on the tarsus of leg IV (Henderickx 1998). The fourth *Lasiochernes* population was found in Pelion in Greece. It was identified as *Lasiochernes jonicus* (Beier 1929, Mahnert 1978), due to the pedipalpal setation of the male specimens, which provides the main character distinguishing *Lasiochernes jonicus* from *Lasiochernes cretonatus*.

The chelicera, palp, leg I and leg IV were removed from the left side of the body of all specimens examined. In the case of *Lasiochernes pilosus*, these appendages were mounted as permanent slide mounts using Swann’s fluid as the medium. The rest of the body was studied as a temporary slide mount using lactic acid, after which it was returned to 70% ethanol. The body and the dissected appendages of *Lasiochernes cretonatus* and *Lasiochernes jonicus* were studied as temporary slide mounts using lactic acid, after which they were returned to 70% ethanol.

**Figure 2.**
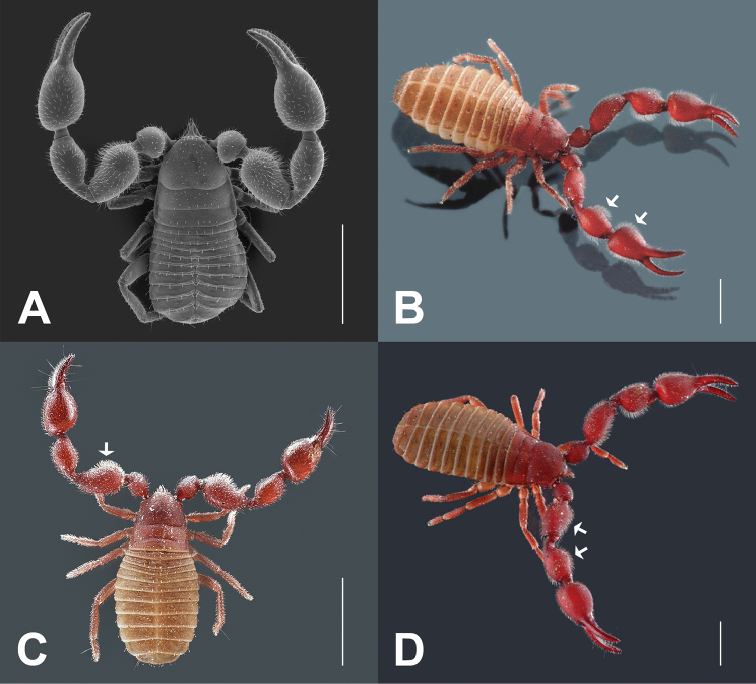
Males of *Lasiochernes* species. **A** *Lasiochernes jonicus* (scanning electron micrograph) **B** *Lasiochernes cretonatus* **C** *Lasiochernes jonicus* **D** *Lasiochernes pilosus*. Arrows point to long, dense setation on palps. Scale lines: 1 mm.

Measurements were taken from photographs using the Zeiss AxioVision 40LE application (v. 4.6). These photographs were made using the Canon EOS Utility software and a digital camera (Canon EOS 1100D) connected to a Zeiss Stemi 2000-C stereomicroscope or a Leica ICC50 camera connected to a Leica DM1000 stereomicroscope using Leica LAS EZ 1.8.0 software. Figures 4, 5 and 6 were drawn using a Leica drawing tube. Figure 2A was made with an FEI Quanta 200 scanning electron microscope at the Royal Belgian Institute of Natural Sciences, Brussels; ESEM scanning was performed in low pressure/low temperature water vapor (100% saturation, 4°C). Figures 2B, C and 2D are photographs of living specimens, taken on a glass plate with flash illumination, using a Canon Eos 5D mark III with a Canon MP-E 65 mm f2.8 lens. Nomenclature for all taxa follows Harvey (2013). The material is deposited in the zoological collections of Comenius University, Bratislava.

Methods of multivariate morphometrics (Marhold 2011) were used to examine the differentiation of 19 adult specimens assigned to three *Lasiochernes* species (five specimens of *Lasiochernes cretonatus*, two specimens of *Lasiochernes jonicus* and 12 specimens of *Lasiochernes pilosus*) and to evaluate the importance of particular morphological characters. The morphological characters measured or scored included those reported as taxonomically relevant within the genus in identification keys and other treatments. The distribution of long and dense setation on the palps of males, the main character used for taxonomic identification of the studied samples, was omitted from the statistical analyses to avoid circular reasoning. Altogether, 92 quantitative characters were measured or scored (Table 1), of which 51 were continuous (see Table 1 in Results) and 34 were discrete (see Morphological descriptions in Results). Out of these, seven characters were invariable between measured specimens (number of blades in cheliceral rallum, number of setae on hand and movable finger of chelicera, number of trichobothria on both chelal fingers, presence of a pair of long tactile setae on tergite XI and sternite XI) and only the remaining 85 characters were used for further statistical analyses.

**Table 1.** Descriptive statistics of the measured morphological characters of the studied *Lasiochernes* species. Abbreviations: n: number of measured specimens. Mean values of the measured characters ± standard deviation (Mean ± SD) are given in upper rows; minimum and maximum (Min–Max) are in lower rows. Values of all the measured characters are in mm.

Characters/Species Mean ± SD Min–Max	*Lasiochernes cretonatus*	*Lasiochernes jonicus*	*Lasiochernes pilosus*			
	Adults	Adults	Adults	Tritonymphs	Deutonymphs	Protonymphs
	n = 5	n = 2	n = 12	n = 15	n = 15	n = 15
Body length	4.23±0.20 4.03–4.51	2.98±1.00 2.27–3.69	3.92±0.65 3.12–4.98	2.73±0.36 2.18–3.38	2.50±0.20 2.11–2.78	1.59±0.13 1.41–1.80
Carapace length	1.01±0.02 0.99–1.03	1.03±0.03 1.01–1.05	1.22±0.08 1.12–1.36	0.97±0.05 0.91–1.09	0.77±0.05 0.69–0.89	0.58±0.04 0.54–0.67
Carapace posterior width	1.00±0.00 0.99–1.00	1.09±0.01 1.08–1.09	1.28±0.13 1.12–1.55	1.04±0.06 0.92–1.13	0.85±0.06 0.73–0.95	0.66±0.04 0.60–0.75
Carapace length/posterior width ratio	1.02±0.02 0.99–1.03	0.95±0.03 0.93–0.97	0.96±0.05 0.88–1.05	0.94±0.03 0.88–0.99	0.91±0.05 0.84–0.99	0.88±0.03 0.83–0.95
Chelicera length	0.35±0.01 0.35–0.36	0.36±0.00 0.36–0.36	0.37±0.04 0.33–0.45	0.28±0.02 0.26–0.31	0.22±0.01 0.21–0.23	0.16±0.01 0.15–017
Chelicera width	0.18±0.01 0.17–0.18	0.17±0.01 0.16–0.18	0.23±0.02 0.20–0.27	0.18±0.01 0.16–0.19	0.13±0.01 0.12–0.14	0.10±0.01 0.09–0.11
Chelicera length/width ratio	2.01±0.07 1.94–2.12	2.13±0.18 2.00–2.25	1.67±0.09 1.54–1.86	1.61±0.07 1.44–1.72	1.72±0.08 1.57–1.83	1.63±0.07 1.55–1.78
Cheliceral movable finger length	0.26±0.01 0.26–0.27	0.21±0.01 0.20–0.21	0.30±0.03 0.25–0.34	0.22±0.07 0.21–0.23	0.17±0.00 0.17–0.18	0.13±0.01 0.12–0.15
Palpal trochanter length	0.52±0.01 0.50–0.53	0.53±0.01 0.52–0.53	0.63±0.06 0.53–0.69	0.42±0.02 0.39–0.45	0.30±0.03 0.27–0.33	0.20±0.01 0.18–0.24
Palpal trochanter width	0.38±0.00 0.38–0.38	0.42±0.02 0.40–0.43	0.43±0.05 0.34–0.51	0.30±0.02 0.27–0.33	0.21±0.01 0.18–0.23	0.14±0.01 0.13–0.15
Palpal trochanter length/width ratio	1.33±0.05 1.27–1.39	1.27±0.05 1.23–1.30	1.46±0.11 1.30–1.63	1.40±0.06 1.29–1.48	1.44±0.11 1.23–1.68	1.42±0.07 1.33–1.60
Palpal femur length	0.95±0.03 0.93–0.99	0.97±0.05 0.93–1.00	1.11±0.09 0.91–1.26	0.72±0.04 0.66–0.79	0.50±0.02 0.47–0.55	0.31±0.02 0.28–0.35
Palpal femur width	0.38±0.01 0.37–0.39	0.50±0.12 0.41–0.58	0.44±0.05 0.38–0.53	0.32±0.02 0.29–0.34	0.22±0.01 0.19–0.25	0.14±0.01 0.12–0.15
Palpal femur length/width ratio	2.50±0.05 2.44–2.54	2.02±0.59 1.60–2.44	2.51±0.19 2.19–2.80	2.29±0.12 2.09–2.48	2.28±0.11 2.17–2.47	2.29±0.14 2.07–2.62
Palpal patella length	0.96±0.03 0.93–0.99	1.02±0.01 1.01–1.02	1.04±0.10 0.82–1.18	0.67±0.04 0.62–0.72	0.46±0.02 0.43–0.48	0.29±0.01 0.27–0.30
Palpal patella width	0.44±0.01 0.42–0.45	0.43±0.01 0.42–0.44	0.49±0.06 0.41–0.60	0.35±0.02 0.32–0.40	0.24±0.01 0.23–0.27	0.15±0.00 0.15–0.16
Palpal patella length/width ratio	2.21±0.06 2.15–2.30	2.36±0.06 2.32–2.40	2.14±0.16 1.90–2.41	1.91±0.08 1.79–2.06	1.87±0.06 1.74–1.96	1.88±0.04 1.80–2.00
Palpal hand with pedicel length	0.89±0.01 0.88–0.91	0.94±0.08 0.88–0.99	1.06±0.10 0.81–1.18	0.77±0.05 0.68–0.83	0.54±0.03 0.51–0.59	0.36±0.02 0.33–0.39
Palpal hand without pedicel length	0.77±0.03 0.74–0.81	0.80±0.06 0.75–0.84	0.93±0.09 0.74–1.05	0.69±0.04 0.60–0.76	0.49±0.03 0.45–0.55	0.32±0.02 0.31–0.37
Palpal hand width	0.58±0.02 0.57–0.61	0.59±0.00 0.59–0.59	0.65±0.06 0.54–0.74	0.47±0.03 0.42–0.52	0.31±0.02 0.28–0.34	0.19±0.01 0.17–0.20
Palpal hand with pedicel length/width ratio	1.53±0.05 1.44–1.58	1.58±0.13 1.49–1.68	1.64±0.08 1.50–1.72	1.53±0.08 1.36–1.65	1.74±0.06 1.64–1.84	1.89±0.14 1.70–2.18
Palpal fixed finger length	0.84±0.06 0.80–0.95	0.74±0.04 0.71–0.77	0.93±0.05 0.83–1.01	0.62±0.04 0.54–0.68	0.43±0.02 0.41–0.48	0.30±0.02 0.27–0.33
Palpal chela length	1.66±0.09 1.58–1.78	1.61±0.07 1.56–1.66	1.93±0.15 1.55–2.12	1.34±0.09 1.20–1.47	0.93±0.03 0.88–0.98	0.63±0.02 0.60–0.69
Palpal chela length/palpal hand width	2.86±0.09 2.77–2.96	2.73±0.12 2.64–2.81	3.00±0.19 2.69–3.36	2.88±0.11 2.71–3.13	2.96±0.13 2.79–3.21	3.35±0.17 3.15–3.65
Leg I trochanter length	0.23±0.02 0.21–0.24	0.22±0.02 0.20–0.23	0.27±0.03 0.23–0.31	0.20±0.01 0.17–0.24	0.13±0.01 0.12–0.15	0.09±0.01 0.08–0.10
Leg I trochanter width	0.17±0.01 0.17–0.18	0.18±0.00 0.18–0.18	0.21±0.02 0.19–0.24	0.16±0.01 0.15–0.19	0.12±0.01 0.11–0.14	0.08±0.01 0.07–0.09
Leg I trochanter length/width ratio	1.31±0.08 1.23–1.41	1.19±0.12 1.11–1.28	1.27±0.08 1.14–1.41	1.24±0.09 1.13–1.40	1.13±0.05 1.07–1.18	1.07±0.06 1.00–1.14
Leg I femur length	0.27±0.01 0.27–0.28	0.26±0.03 0.24–0.28	0.31±0.03 0.25–0.35	0.20±0.02 0.17–0.23	0.13±0.01 0.12–0.15	0.10±0.01 0.09–0.12
Leg I femur width	0.17±0.01 0.17–0.18	0.19±0.01 0.18–0.20	0.23±0.02 0.20–0.25	0.16±0.01 0.14–0.20	0.11±0.01 0.10–0.13	0.08±0.01 0.07–0.11
Leg I femur length/width	1.58±0.07 1.50–1.65	1.37±0.05 1.33–1.40	1.37±0.08 1.24–1.50	1.23±0.08 1.13–1.43	1.17±0.11 1.00–1.40	1.19±0.08 1.00–1.29
Leg I patella length	0.50±0.06 0.44–0.58	0.48±0.01 0.47–0.48	0.55±0.04 0.46–0.61	0.38±0.02 0.34–0.41	0.27±0.02 0.25–0.30	0.18±0.01 0.16–0.19
Leg I patella width	0.17±0.02 0.15–0.19	0.16±0.00 0.16–0.16	0.20±0.02 0.17–0.22	0.15±0.01 0.13–0.17	0.11±0.01 0.10–0.12	0.08±0.01 0.07–0.09
Leg I patella length/width ratio	3.03±0.20 2.75–3.22	2.97±0.04 2.94–3.00	2.78±0.19 2.42–3.06	2.57±0.12 2.33–2.67	2.57±0.14 2.36–2.80	2.26±0.13 2.11–2.57
Leg I tibia length	0.52±0.06 0.46–0.60	0.47±0.04 0.44–0.49	0.55±0.05 0.46–0.62	0.36±0.02 0.33–0.41	0.24±0.01 0.23–0.26	0.16±0.01 0.15–0.17
Leg I tibia width	0.13±0.01 0.12–0.15	0.12±0.01 0.11–0.12	0.15±0.01 0.13–0.16	0.11±0.02 0.10–0.13	0.08±0.00 0.08–0.09	0.06±0.00 0.06–0.07
Leg I tibia length/width	3.97±0.21 3.73–4.29	4.04±0.06 4.00–4.08	3.81±0.29 3.44–4.21	3.23±0.15 3.00–3.50	2.88±0.10 2.67–3.00	2.55±0.15 2.29–2.83
Leg I tarsus length	0.42±0.05 0.38–0.47	0.33±0.03 0.31–0.35	0.49±0.04 0.42–0.56	0.35±0.02 0.31–0.38	0.25±0.01 0.23–0.26	0.17±0.01 0.15–0.19
Leg I tarsus width	0.11±0.01 0.10–0.12	0.09±0.01 0.08–0.09	0.11±0.01 0.09–0.12	0.09±0.01 0.08–0.09	0.07±0.01 0.06–0.07	0.05±0.00 0.05–0.06
Leg I tarsus length/width ratio	3.88±0.52 3.25–4.70	3.91±0.66 3.44–4.38	4.48±0.40 3.83–5.11	4.02±0.19 3.67–4.38	3.77±0.20 3.57–4.17	3.33±0.21 3.00–3.60
Leg IV trochanter length	0.39±0.02 0.37–0.42	0.33±0.04 0.30–0.35	0.43±0.06 0.34–0.53	0.33±0.01 0.30–0.35	0.21±0.02 0.20–0.24	0.13±0.01 0.10–0.15
Leg IV trochanter width	0.20±0.01 0.19–0.21	0.18±0.01 0.17–0.19	0.26±0.03 0.21–0.29	0.21±0.01 0.19–0.22	0.14±0.01 0.12–0.16	0.09±0.01 0.08–0.11
Leg IV trochanter length/width ratio	1.91±0.12 1.81–2.10	1.80±0.05 1.76–1.84	1.69±0.13 1.48–1.91	1.61±0.06 1.55–1.75	1.59±0.10 1.40–1.71	1.48±0.15 1.11–1.67
Leg IV femoropatella length	0.81±0.05 0.74–0.85	0.91±0.09 0.84–0.97	1.04±0.09 0.88–1.18	0.71±0.04 0.63–0.76	0.51±0.02 0.48–0.54	0.33±0.02 0.30–0.35
Leg IV femoropatella width	0.19±0.02 0.17–0.21	0.19±0.01 0.18–0.19	0.23±0.03 0.19–0.27	0.20±0.01 0.18–0.23	0.15±0.01 0.13–0.16	0.10±0.01 0.09–0.11
Leg IV femoropatella length/width ratio	4.26±0.34 3.76–4.72	4.89±0.31 4.67–5.11	4.51±0.33 4.00–4.95	3.67±0.18 3.30–3.89	3.51±0.14 3.27–3.77	3.39±0.13 3.10–3.56
Leg IV tibia length	0.76±0.03 0.74–0.80	0.72±0.03 0.70–0.74	0.84±0.08 0.71–0.96	0.56±0.03 0.50–0.60	0.37±0.02 0.35–0.40	0.23±0.01 0.21–0.25
Leg IV tibia width	0.13±0.00 0.12–0.13	0.14±0.00 0.14–0.14	0.15±0.02 0.12–0.17	0.13±0.01 0.12–0.15	0.11±0.01 0.10–0.11	0.08±0.01 0.07–0.08
Leg IV tibia length/width	5.97±0.28 5.69–6.33	5.14±0.20 5.00–5.29	5.57±0.45 4.44–6.33	4.29±0.32 3.79–4.75	3.53±0.09 3.36–3.70	3.06±0.12 2.88–3.29
Leg IV tarsus length	0.48±0.02 0.46–0.50	0.40±0.03 0.38–0.42	0.57±0.04 0.49–0.64	0.41±0.02 0.38–0.44	0.29±0.01 0.27–0.31	0.20±0.01 0.18–0.21
Leg IV tarsus width	0.11±0.00 0.11–0.11	0.10±0.01 0.09–0.10	0.12±0.01 0.09–0.14	0.10±0.01 0.09–0.11	0.08±0.01 0.07–0.09	0.06±0.00 0.05–0.06
Leg IV tarsus length/width ratio	4.40±0.15 4.18–4.55	4.23±0.61 3.80–4.67	4.80±0.43 4.17–5.70	4.02±0.21 3.64–4.44	3.60±0.25 3.11–4.00	3.46±0.18 3.00–3.80

The statistical analyses were performed as follows:

(1) As the first step, the Shapiro-Wilk statistic for the test of normality of distribution was computed for each character.

(2) Principal coordinate analysis, PCoA (Podani 2000, 2001), based on 85 characters, was used to obtain possible groupings of the 19 studied specimens. The data were standardized by a standard deviation of variables, and Euclidean distance was used to compute the secondary matrix. PCoA, unlike the better known PCA method (principal component analysis), can be also used for qualitative and mixed characters, as well as in cases when p>n (p = number of characters, n = number of objects).

(3) Correlation between the principal coordinate axes of PCoA and original quantitative characters was computed using Pearson correlation coefficient (Zar 1999) in order to identify the characters that are the most responsible for the groupings of specimens along the first three principal coordinate axes.

(4) Discriminant analyses (Klecka 1980, Marhold 2011) were employed to assess the morphological differentiation between the three *Lasiochernes* species. The discriminant analyses applied included canonical discriminant analysis (CDA) and classificatory discriminant analysis (classificatory DA). In CDA, the discriminant functions were derived to express the extent of morphological differentiation between the predefined groups (the three *Lasiochernes* species) and to identify the most important differentiating characters. Nonparametric *k*-nearest neighbors classificatory discriminant analyses were performed to estimate the percentage of specimens correctly assigned to the predefined groups. A cross-validation procedure was used, in which the classification criterion was based on *n*−1 individuals and then applied to the individual left out. Discriminant analyses generally require a multivariate normal distribution of the characters; nevertheless, they have been shown to be quite robust against deviations in this respect (Thorpe 1976, Klecka 1980). Due to the limited number of available specimens (19) and the chosen number of predefined groups (three), we had to lower the number of characters in primary matrices to 15 (or less) in order to satisfy the requirements for number of objects (n), number of predefined groups (g) and number of variables (p) in discriminant analyses [p < (n−g)]. Therefore, the original dataset of all measured characters was divided into eight partial matrices corresponding to eight parts of the body. Each partial dataset contained no more than 15 characters and each was analyzed in a separate CDA and classificatory DA. The following eight body parts were selected: carapace (six characters), chelicera (six characters), palp (nine characters), chela (11 characters), leg I (15 characters), leg IV (12 characters), tergites (ten characters) and sternites (12 characters). As a result, eight CDAs (CDA 1–CDA 8) and eight classificatory DAs (DA 1–DA 8) were performed to identify both the body parts and the characters that are most important for the differentiation of the three species. Altogether, 81 characters (out of the original 85) were included in these analyses. Four characters were omitted. The character “length of the whole body” was inapplicable for the parts of the body and three other characters (posterior width of carapace, length of palpal hand with pedicel, length of patella of leg I), were excluded because they were invariable within one or more predefined groups (species) and might have distorted the discriminant analyses. Based on the results of the eight CDAs (CDA 1–8), the 15 most important characters were selected and a final matrix, combining all body parts, was assembled. This total-body matrix was analyzed in CDA 9 and classificatory DA 9. Prior to the discriminant analyses of all the datasets mentioned above, the Pearson and nonparametric Spearman correlation coefficients (Zar 1999) were computed to reveal correlation structure among the selected characters and to ensure that no very high correlations (> 0.95) were present (potentially distorting the analyses). The discriminant analyses were performed using SAS 9.1.3 software SAS/STAT v.9.2 (SAS Institute 2009).

(5) Finally, descriptive statistics were computed for adults of the three *Lasiochernes* species, and for nymphs of *Lasiochernes pilosus*. Variations in the morphological characters that differentiate between them are shown as box-and-whisker plots. The minimum and maximum values for the measured characters are reported in identification key and morphological descriptions. The analyses were performed using SAS 9.1.3 software SAS/STAT v.9.2 (SAS Institute 2009).

## Results

**Figure 3.**
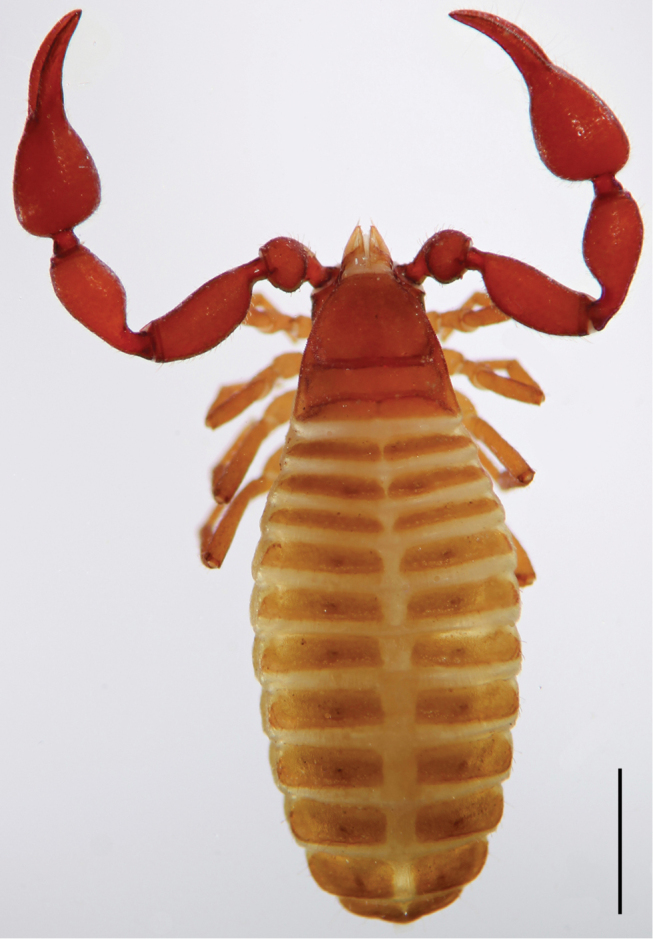
Female of *Lasiochernes cretonatus*. Scale line: 1 mm.

**Figure 4.**
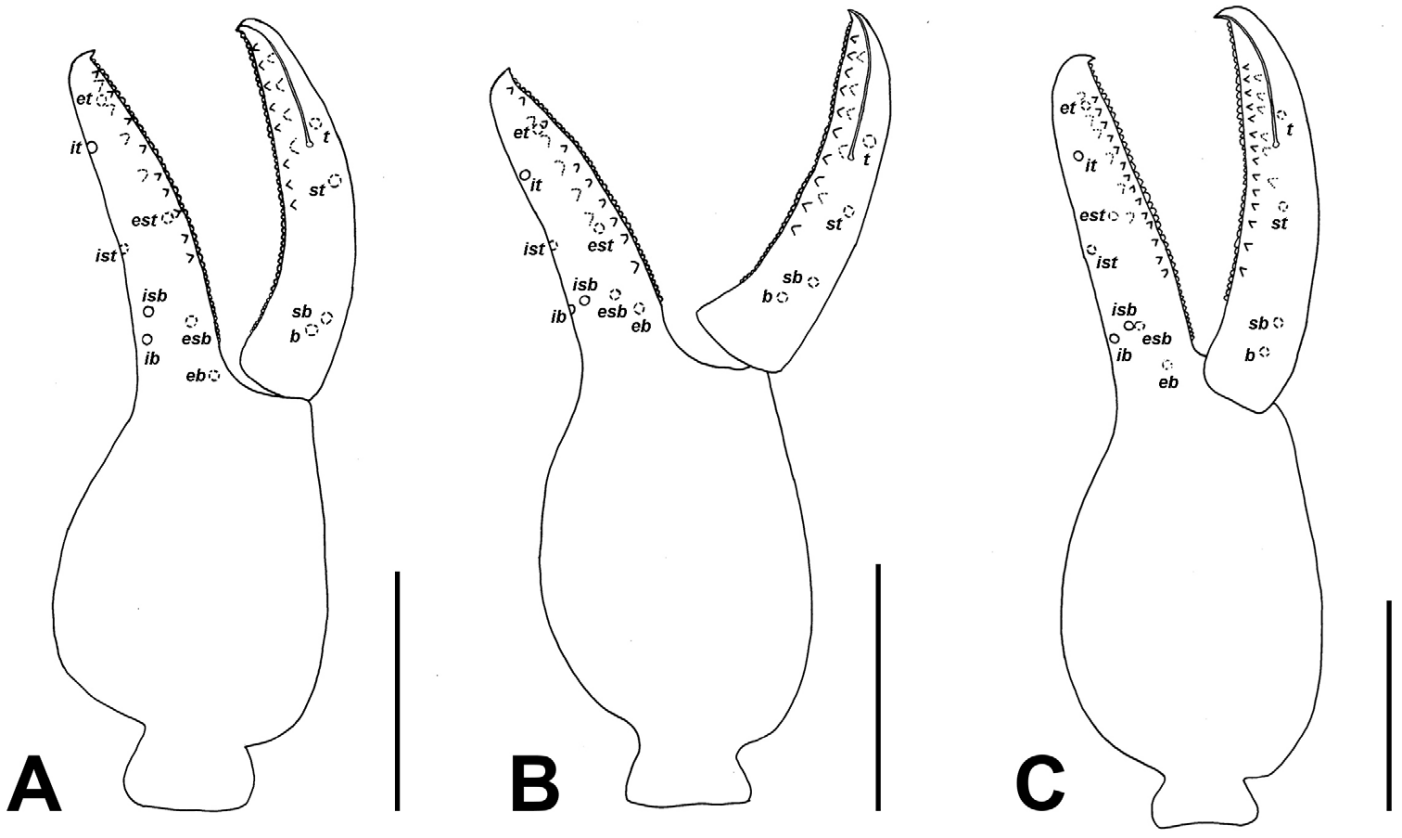
Palpal chela of *Lasiochernes* species, showing the trichobothrial pattern. **A** *Lasiochernes cretonatus* male **B** *Lasiochernes jonicus* female **C** *Lasiochernes pilosus* male. Abbreviations in terminology of trichobothria: movable finger: *t*–terminal, *st*–subterminal, *sb*–subbasal, *b*–basal; fixed finger: *et*–exterior terminal, *est*–exterior subterminal, *esb*–exterior subbasal, *eb*–exterior basal, *it*–interior terminal, *ist*–interior subterminal, *isb*–interior subbasal, *ib*–interior basal. Scale lines: 0.5 mm.

*Morphological descriptions*. Adults of the studied *Lasiochernes* species share the following characteristic. Setae on body relatively short and clavate. Carapace approximately as long as broad, granulate and rectangular, epistome absent, anterior margin straight, eyes or eyespots absent, anterior and posterior transverse furrows distinct (Figs 2A, 3). Chelicerae small, slightly sclerotized, five setae on hand, one on movable finger; movable finger with slender, well-developed galea; rallum of three blades; small, largely unsclerotized teeth situated on both movable and fixed fingers. Palps (Fig. 4): chelal fingers with twelve trichobothria (eight on fixed and four on movable chelal finger), venom apparatus developed only in movable chelal finger. Legs: tarsus IV with long tactile seta (Fig. 2). Abdominal tergites divided, tergite XI with a pair of long tactile setae (Fig. 2). Body measurements are given in Table 1.

### Lasiochernes cretonatus

Taxon classificationAnimaliaPseudoscorpionesChernetidae

Henderickx, 1998

Figs 2B , 3 ; Table 1

#### Description.

**Female (4 specimens analyzed)** (Table 1). Chaetotaxy of carapace: 71–74 setae, 31–38 of them situated in front of anterior transverse furrow, 21–26 on medial disk, posterior margin with 13–14 setae. Cheliceral galea with 5–6 short terminal rami, serrula exterior with 19–21 blades. Palps: fixed chelal finger with 44–48 and movable chelal finger with 48–50 marginal teeth; fixed chelal finger with 9–13 antiaxial accessory teeth and movable chelal finger with 8–9 antiaxial accessory teeth; fixed and movable chelal fingers with four paraxial accessory teeth. Palpal parts without long, dense setation (Fig. 3). Legs: tarsus IV with long tactile seta situated one third from the joint with the tibia, meaning 0.15–0.19 mm from the tarsal base. Chaetotaxy of tergites I–X: 14–16 (left hemitergite 6–8 + right hemitergite 7–8): 14–17 (7–8 + 7–9): 13–18 (7–9 + 6–9): 19–24 (9–11 + 9–13): 21–25 (11–13 + 10–12): 18–27 (9–15 + 9–12): 19–23 (10–11 + 9–12): 21–22 (10–12 + 10–11): 19–22 (10–12 + 9–12): 14–18 (7–9 + 7–9); tergite XI with 10 setae (5 + 5) plus a pair of long tactile setae. Chaetotaxy of sternites IV–X: 8–13 (left hemisternite 4–6 + right hemisternite 4–8): 18–22 (9–11 + 9–11): 20–25 (10–12 + 10–13): 19–23 (9–11 + 9–12): 19–26 (10–12 + 9–14): 22–24 (10–12 + 11–13): 18–22 (9–11 + 9–12); sternite XI with 9–10 setae (4–5 + 5) plus a pair of long tactile setae. Female spermatheca unpaired, T-shaped; anterior genital operculum with 29–31 setae and two lyrifissures, posterior operculum with 10–12 setae and 4–6 lyrifissures (Fig. 6A).

**Male (1 specimen analyzed)** (Fig. 2B, Table 1). Chaetotaxy of carapace: 82 setae, 42 of them on anterior disk, 27 on medial disk, posterior margin with 13 setae. Cheliceral galea with six short terminal rami, serrula exterior with 20 blades. Palps (Fig. 4A): fixed chelal finger with 44 and movable chelal finger with 49 marginal teeth; fixed chelal finger with nine and movable chelal finger with eight antiaxial accessory teeth; fixed and movable chelal fingers with four paraxial accessory teeth. Palpal hand and patella with long and dense setation on their medial sides (Fig. 2B). Legs: tarsus IV with long tactile seta situated one third from the joint with the tibia, that means 0.16 mm from the tarsal base. Chaetotaxy of tergites I–XI: 16 (left hemitergite 9 + right hemitergite 7): 17 (8 + 9): 18 (9 + 9): 24 (12 + 12): 24 (13 + 11): 21 (11 + 10): 21 (10 + 11): 21 (10 + 11): 22 (12 + 10): 21 (10 + 11), tergite XI with 10 setae (5 + 5) and with a pair of long tactile setae. Chaetotaxy of sternites IV–XI: 14 (left hemisternite 8 + right hemisternite 6): 25 (12 + 13): 26 (12 + 14): 25 (13 + 12): 26 (13 + 13): 23 (11 + 12): 22 (11 + 11), sternite XI with 11 (5 + 6) and with a pair of long tactile setae. Anterior genital operculum with 50 setae and two lyrifissures, posterior operculum with 20 setae and six lyrifissures (Fig. 6B).

### Lasiochernes jonicus

Taxon classificationAnimaliaPseudoscorpionesChernetidae

(Beier, 1929)

Figs 2A, C ; Table 1

#### Description.

**Female (1 specimen analyzed)** (Table 1). Chaetotaxy of carapace: 93 setae, 51 of them situated on anterior disk, 28 on medial disk, posterior margin with 14 setae. Cheliceral galea with six short terminal rami, serrula exterior with 20 blades. Palps (Fig. 4B): fixed chelal finger with 44 and movable chelal finger with 49 marginal teeth; fixed and movable chelal fingers with ten antiaxial accessory teeth and with five paraxial accessory teeth. Palpal femur with normal shape and without long and dense setation. Legs: tarsus IV with long tactile seta situated near middle of segment, that means 0.21 mm from the tarsal base. Chaetotaxy of tergites I–XI: 14 (left hemitergite 6 + right hemitergite 8): 14 (7 + 7): 14 (7 + 7): 19 (9 + 10): 21 (11 + 10): 19 (9 + 10): 19 (10 + 9): 20 (11 + 9): 17 (9 + 8): 17 (8 + 9), tergite XI with 8 setae (4 + 4) and with a pair of long tactile setae. Chaetotaxy of sternites IV–XI: 9 (left hemisternite 5 + right hemisternite 4): 21 (11 + 10): 24 (11 + 13): 26 (13 + 13): 26 (12 + 14): 23 (12 + 11): 17 (9 + 8), sternite XI with 8 (4 + 4) and with a pair of long tactile setae. Female spermatheca unpaired, T-shaped; anterior genital operculum with 34 setae and two lyrifissures, posterior operculum with 12 setae and three lyrifissures (Fig. 6C).

**Male (1 specimen analyzed)** (figs 2A, 2C; Table 1). Carapace with 82 setae, 37 of them on anterior disk, 32 on medial disk, posterior margin with 13 setae. Cheliceral galea with five short terminal rami, serrula exterior with 21 blades. Palps: fixed chelal finger with 42 and movable chelal finger with 47 marginal teeth; fixed chelal finger with 12 antiaxial and movable chelal finger with ten antiaxial accessory teeth; fixed chelal finger with six paraxial and movable finger with four paraxial accessory teeth. Palpal femur basally markedly broad, on the medial side with long and dense setation (Figs 2A, 2C). Legs: tarsus IV with long tactile seta situated near the middle of segment, that means 0.19 mm from the tarsal base Chaetotaxy of tergites I–XI: 15 (left hemitergite 7 + right hemitergite 8): 14 (7 + 7): 15 (7 + 8): 18 (9 + 9): 17 (10 + 7): 17 (8 + 9): 17 (9 + 8): 19 (10 + 9): 18 (9 + 9): 13 (7 + 6), tergite XI with 8 setae (4 + 4) and with a pair of long tactile setae. Chaetotaxy of sternites IV–XI: 25 (left hemisternite 13 + right hemisternite 12): 29 (15 + 14): 25 (12 + 13): 25 (12 + 13): 26 (13 + 13): 22 (10 + 12): 18 (9 + 9), sternite XI with 9 (4 + 5) and with a pair of long tactile setae. Anterior genital operculum with 48 setae and two lyrifissures, posterior operculum with 31 setae and ten lyrifissures (Fig. 6D).

### Lasiochernes pilosus

Taxon classificationAnimaliaPseudoscorpionesChernetidae

(Ellingsen, 1910)

Fig. 2D ; Table 1

#### Description.

**Female (7 specimens analyzed)** (Table 1). Chaetotaxy of carapace: 81–96 setae, 49–63 of them situated on anterior disk, 17–25 on medial disk, posterior margin with 10–13 setae. Cheliceral galea with 6–8 short terminal rami, serrula exterior with 23–25 blades. Palps: fixed chelal finger with 44–49 and movable chelal finger with 44–49 marginal teeth; fixed chelal finger with 11–16 antiaxial and movable chelal finger with 11–15 antiaxial accessory teeth; fixed and movable chelal finger with 6–7 paraxial accessory teeth. Palpal parts without long and dense setation. Legs: tarsus IV with long tactile seta situated approximately in the middle of segment, that means 0.25–0.32 mm from the tarsal base. Chaetotaxy of tergites I–XI: 12–17 (left hemitergite 6–9 + right hemitergite 6–8): 15 (7–8 +7–8): 14–19 (8–9 + 6–10): 17–24 (7–11+ 9–13): 18–23 (9–11 + 9–12): 18–22 (8–12 + 8–11): 18–23 (8–12 + 9–11): 18–22 (9–11 + 9–11): 17–20 (8–11 + 7–10): 13–19 (6–10 + 6–9), tergite XI with 8 setae (4 + 4) and with a pair of long tactile setae. Chaetotaxy of sternites IV–XI: 8–18 (left hemisternite 4–10: right hemisternite 4–9): 17–25 (9–13 + 8–13): 19–28 (9–13 + 10–15): 19–28 (9–15 + 9–13): 17–27 (9–13 + 8–14): 18–26 (8–13 + 9–13): 17–22 (8–11 + 8–11), sternite XI with 8–14 (4–6 + 4–5) and with a pair of long tactile setae. Female spermatheca unpaired, T-shaped; anterior genital operculum with 29–44 setae and 1–2 lyrifissures, posterior operculum with 10–14 setae and 1–4 lyrifissures (Fig. 6E).

**Male (5 specimens analyzed)** (Fig. 2D, Table 1). Chaetotaxy of carapace: 77–89 setae, 47–57 of them on anterior disk, 18–23 on medial disk, posterior margin with 10–14 setae. Cheliceral galea with 6–7 short terminal rami, serrula exterior with 23–24 blades. Palps (Fig. 4C): fixed chelal finger with 40–50 and movable chelal finger with 41–51 marginal teeth; fixed chelal finger with 12–16 antiaxial and movable chelal finger with 13–15 antiaxial accessory teeth; fixed chelal finger with 6–7 paraxial and movable chelal finger with six paraxial accessory teeth. Palpal femur and patella with long and dense setation on their medial sides (Fig. 2D). Legs: tarsus IV with long tactile seta situated approximately in the middle of segment, that means 0.25–0.31 mm from the tarsal base. Chaetotaxy of tergites I–XI: 13–17 (left hemitergite 7–9 + right hemitergite 6–8): 14–16 (7–8 + 7–8): 15–22 (7–11 + 8–11): 18–24 (10–12 + 7–13): 19–24 (10–12 + 9–12): 18–22 (9–12 + 9–12): 16–22 (7–10 + 9–12): 17–22 (9–11 + 8–11): 15–19 (8–9 + 7–10): 10–15 (5–7 + 5–8), tergite XI with 8 setae (4 + 4) and with a pair of long tactile setae. Chaetotaxy of sternites IV–XI: 16–24 (left hemisternite 8–11 + right hemisternite 7–13): 17–26 (9–16 + 8–12): 17–31 (6–15 + 11–16): 14–30 (2–15 + 12–15): 22–29 (10–17 + 9–13): 19–27 (9–14 + 9–13): 16–22 (8–12 + 8–11), sternite XI with 8–12 (4–6 + 4–6) and with a pair of long tactile setae. Anterior genital operculum with 44–62 setae and 1–2 lyrifissures, posterior operculum with 19–26 setae and 2–6 lyrifissures (Fig. 6F).

**Figure 5.**
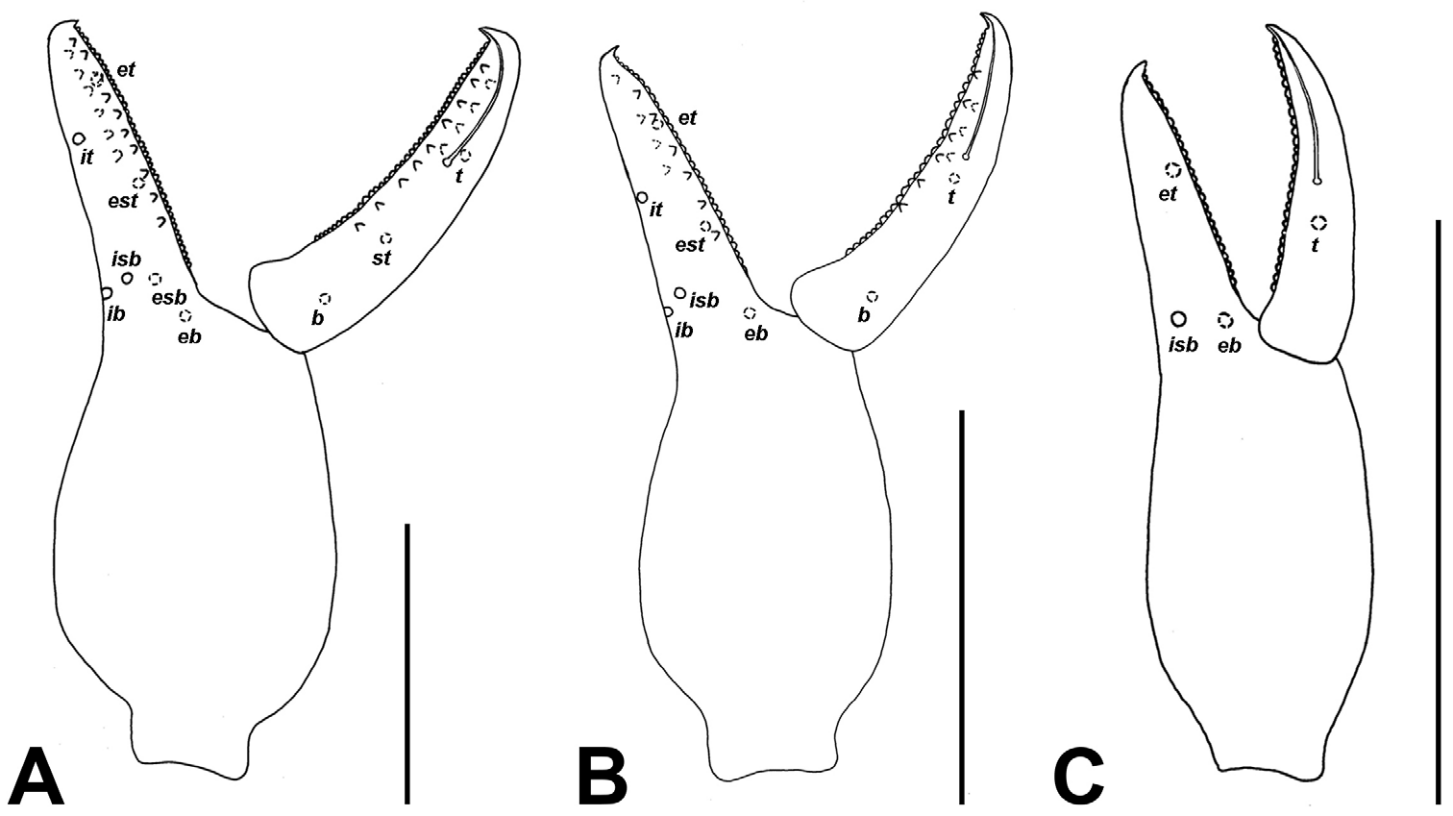
Palpal chela of *Lasiochernes pilosus* nymphs, showing the trichobothrial pattern. **A** Tritonymph **B** Deutonymph **C** Protonymph. Abbreviations as for Figure 4. Scale lines: 0.5 mm.

**Nymphs** (Fig. 5; Table 1): The morphology of tritonymphs, deutonymphs and protonymphs is similar in most respects to that of adults (e.g. morphology of setae on body, granulation of carapace, cheliceral rallum of three blades, presence of venom apparatus in movable chelal finger (Fig. 5), presence of a pair of relatively long tactile setae on tergite XI and long tactile seta situated approximately in the middle of leg IV tarsus). Body measurements are given in Table 1.

**Tritonymphs (15 specimens analyzed)** (Table 1). Chaetotaxy of carapace: 71–87 setae, 43–52 of them situated on anterior disk, 17–25 on medial disk, posterior margin with 9–11 setae. Chelicera: five setae on hand, one on movable finger; galea with six short terminal rami, serrula exterior with 18–20 blades. Palps (Fig. 5A): seven trichobothria on fixed chelal finger and three on movable chelal finger; fixed chelal finger with 34–42 and movable chelal finger with 36–41 marginal teeth; fixed chelal finger with 8–11 antiaxial and movable chelal finger with 8–12 antiaxial accessory teeth; fixed chelal finger with 4–6 paraxial and movable chelal finger with 4–5 paraxial accessory teeth. Chaetotaxy of tergites I–X: 10–13 (left tergite half 5–6 + right tergite half 5–7): 10–12 (5–7 + 5–6): 10–14 (5–7 + 5–8): 11–17 (5–8 + 6–9): 12–17 (6–9 + 6–8): 11–17 (5–8 + 6–9): 13–18 (6–9 + 6–10): 12–17 (5–9 + 6–8): 11–15 (5–8 + 5–7): 9–12 (4–6 + 4–6), tergite XI with 6 setae (3 + 3) and a pair of long tactile setae. Chaetotaxy of sternites II–X: 4–12 (left hemisternite 2–6 + right hemisternite 2–6): 5–11 (2–6 + 3–6): 8–13 (4–7 + 3–7): 12–18 (5–9 + 5–10): 14–19 (7–10 + 7–10): 14–17 (7–10 + 5–9): 13–18 (6–10 + 7–10): 14–18 (7–9 + 7–9), 12–17 (6–8 + 6–9), sternite XI with 8–10 (4–5 + 4–5) and a pair of long tactile setae; sternites II with two lyrifissures.

**Deutonymphs (15 specimens analyzed)** (Table 1). Chaetotaxy of carapace: 44–58 setae, 28–34 of them ivesituated on anterior disk, 9–20 on medial disk, posterior margin with 6–8 setae. Chelicera: five setae on hand, one on movable finger; galea with 3–4 short terminal rami, serrula exterior with 17–19 blades. Palps (Fig. 5B): six trichobothria on fixed chelal finger and two on movable chelal finger; fixed chelal finger with 27–32 and movable chelal finger with 29–33 marginal teeth; fixed chelal finger with 5–7 antiaxial and movable chelal finger with 5–7 antiaxial accessory teeth; fixed chelal finger with 4–5 paraxial and movable chelal finger with three paraxial accessory teeth. Chaetotaxy of tergites I–X: 8–10 (left tergite half 4–5 + right tergite half 4–5): 7–10 (3–5 + 3–5): 6–10 (3–5 + 1–5): 9–10 (4–5 + 4–5): 9–10 (4–5 + 5): 7–10 (3–5 + 3–5): 9–10 (4–5 + 4–5): 9–10 (4–5 + 4–5): 8–10 (4–5 + 4–5): 4–9 (3–5 + 1–5), tergite XI with 4 setae (2 + 2) and a pair of long tactile setae. Chaetotaxy of sternites II–X: 0–1 (left hemisternite 0–0 + right hemisternite 0–1): 4–6 (2–3 + 2–3): 5–8 (2–4 + 2–4): 6–11 (3–6 + 3–5): 7–12 (4–6 + 3–6): 9–11 (4–6 + 4–6): 8–10 (5 + 3–5): 9–11 (4–6 + 4–6), 8–10 (4–5 + 3–5), sternite XI with 6–7 (3–4 + 3–4) and a pair of long tactile setae; sternites II with two lyrifissures.

**Protonymphs (15 specimens analyzed)** (Table 1). Chaetotaxy of carapace: 29–38 setae, 17–22 of them on anterior disk, 4–11 on medial disk, posterior margin with 6–8 setae. Chelicera: four setae on hand, none on movable finger; galea with 3–4 short terminal rami, serrula exterior with 11–14 blades. Palps (Fig. 5C): three trichobothria on fixed chelal finger and 1 trichobothrium on movable chelal finger; fixed chelal finger with 24–29 and movable chelal finger with 26–31 marginal teeth; both chelal finger without any accessory teeth. Chaetotaxy of tergites I–X: each with 6 setae (left tergite half 3 + right tergite half 3), tergite XI with 2 setae (1 + 1) and a pair of long tactile setae. Chaetotaxy of sternites II–X: 0–9 (left hemisternite 0–1 + right hemisternite 0–9): 2 (1 + 1): 3–5 (1–3 + 1–3): 6–8 (3–5 + 3): 6–7 (3 + 3–4): 6–7 (3–4 + 3): 4–7 (3+ 1–4): 5–6 (2–3 + 3), 4–6 (2–3 + 2–3), sternite XI with 2 (1 + 1) and a pair of long tactile setae; sternites II with two lyrifissures.

### Multivariate morphometrics

Most of the measured characters showed departures from a normal distribution. Therefore, the nonparametric correlation coefficient (Spearman) (apart from the Pearson parametric coefficient) and nonparametric classificatory discriminant analyses were used.

The ordination diagram of PCoA of the three *Lasiochernes* species, based on 85 morphological characters for 19 adult specimens, showed two large groupings of specimens separated along the first principal coordinate axis (Fig. 7). The first grouping consisted of *Lasiochernes pilosus* specimens and the second comprised both *Lasiochernes cretonatus* and *Lasiochernes jonicus*. However, the specimens of the latter two species were not intermingled, being divided in accordance with their taxonomic assignment along the second and partly the third principal coordinate axis. The calculations of the correlation between the principal coordinate axes of PCoA and the original quantitative characters revealed the characters most responsible for the grouping of specimens along the first three axes. The characters most correlated with the first axes are: carapace length, length and width of femur of leg I, length of femoropatella of leg IV, length of palpal hand with and without pedicel, chelicera width, width of trochanter of leg I, posterior width of carapace and length of trochanter of leg I. The characters most correlated with the second axis are: numbers of setae on sternite X, tergite VIII, tergite VII, tergite VI and sternite IX; and those most correlated with the third axis are: body length, number of setae on anterior and posterior genital opercula, length/width ratio of tibia of leg IV and number of setae on sternite IV.

**Figure 6.**
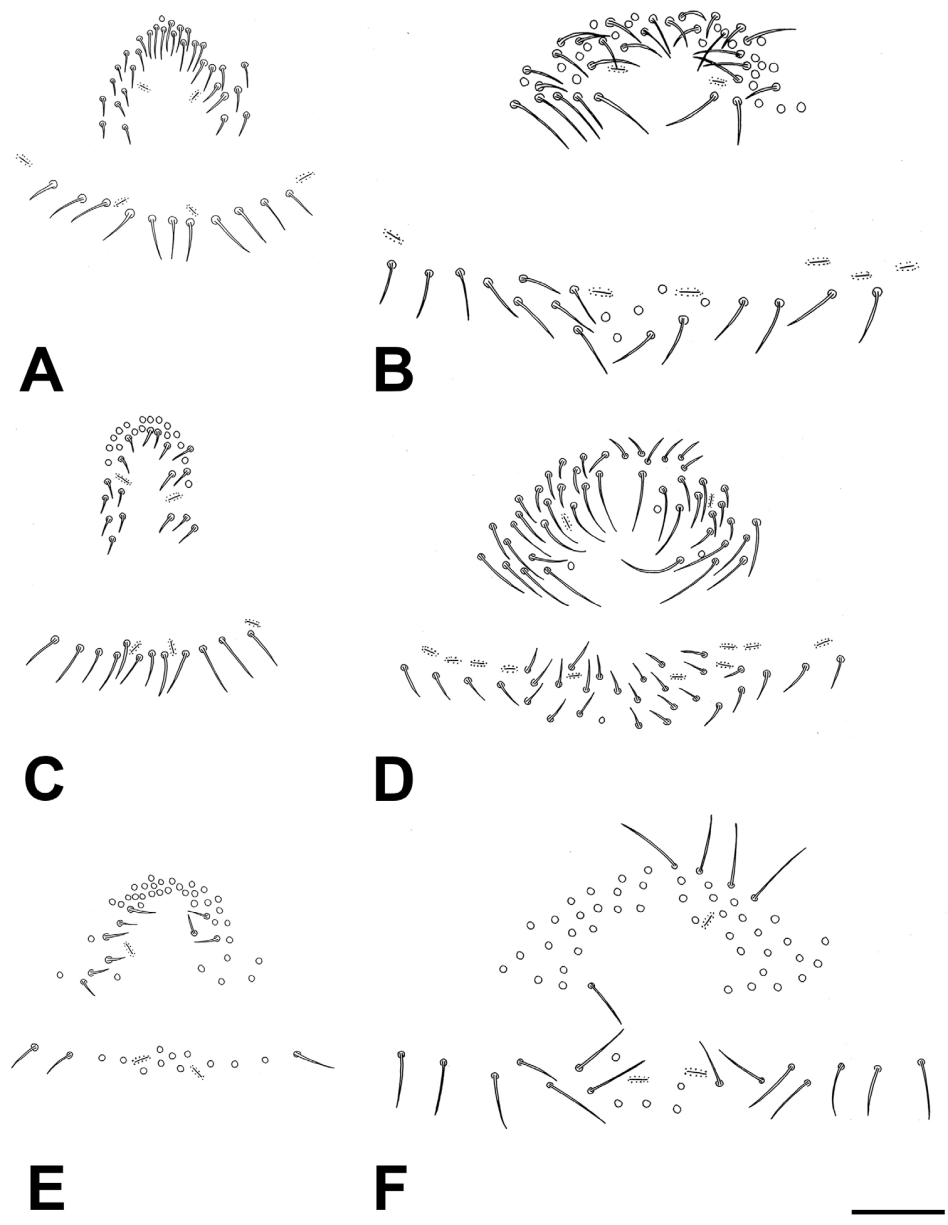
Variation in the setation of the genital area of *Lasiochernes* adults. **A** Female of *Lasiochernes cretonatus* **B** Male of *Lasiochernes cretonatus* **C** Female of *Lasiochernes jonicus* **D** Male of *Lasiochernes jonicus* **E** Female of *Lasiochernes pilosus* **F** Male of *Lasiochernes pilosus*. Scale lines: 0.1 mm.

**Figure 7.**
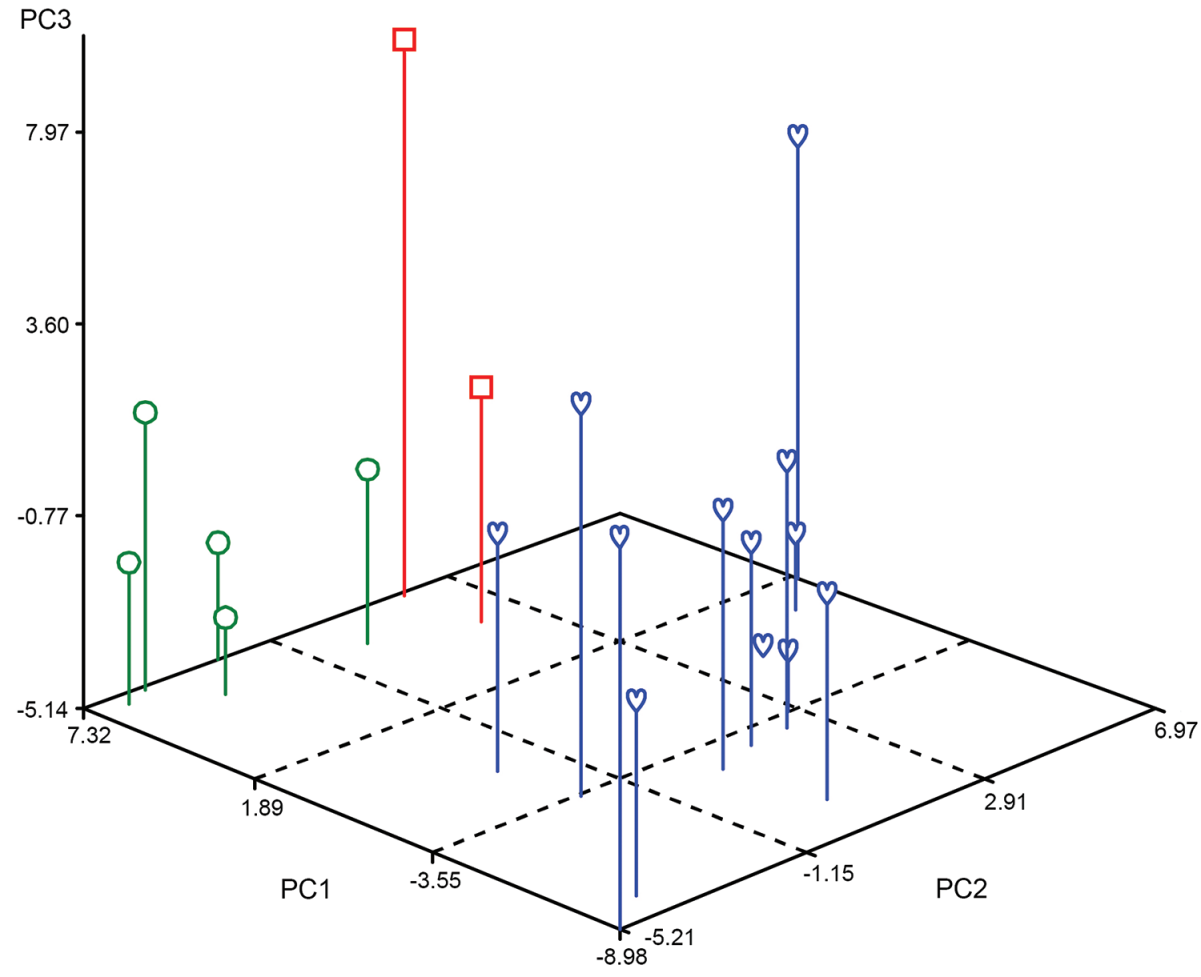
Principal coordinate analysis (PCoA) of 19 adult specimens of three species of *Lasiochernes* based on 85 morphological characters: *Lasiochernes cretonatus* (green circles), *Lasiochernes jonicus* (red squares) and *Lasiochernes pilosus* (blue hearts). The first three coordinate axes explain 37.8%, 15.1% and 12.6% of the variation.

**Figure 8.**
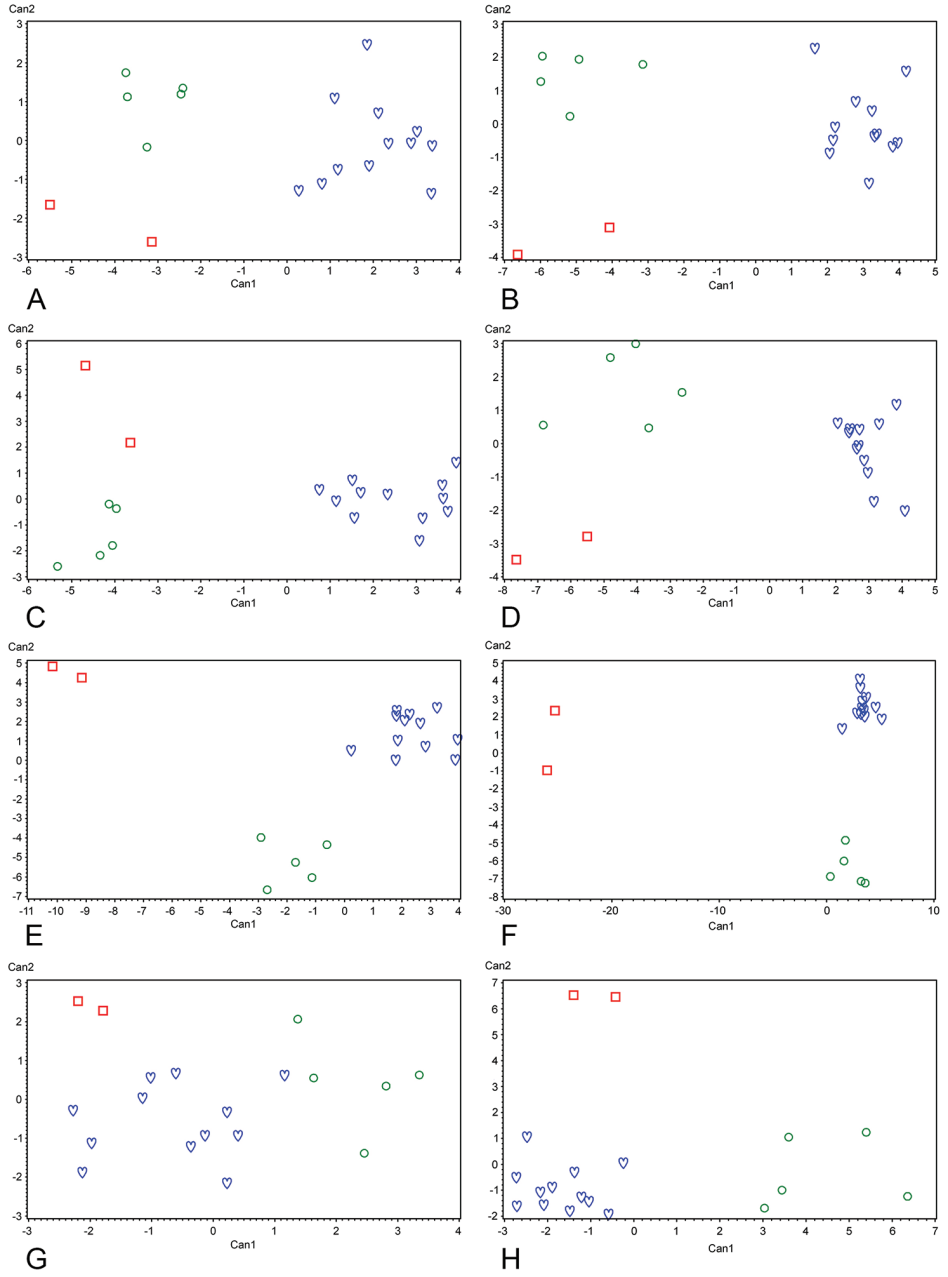
Eight canonical discriminant analyses (CDA 1–8) of three *Lasiochernes* species (*Lasiochernes cretonatus*: green circles; *Lasiochernes jonicus*: red squares; *Lasiochernes pilosus*: blue hearts) based on 19 adult specimens and morphological characters measured/scored on eight different parts of the body (**A–G**): **A** CDA 1: Carapace **B** CDA 2: Chelicera **C** CDA 3: Palp (without chela) **D** CDA 4: Chela **E** CDA 5: Leg I **F** CDA 6: Leg IV **G** CDA 7: Tergites **H** CDA 8: Sternites. For total canonical structure and the lists of characters measured/scored on each body parts, see Supplementary file 1.

Eight canonical (CDA 1–CDA 8) and classificatory discriminant analyses (DA 1–DA 8) were performed to identify the characters and body parts that are most important for the differentiation of the three species, and to evaluate the degree of differentiation in each case. The three character pairs (length and posterior width of carapace, length of palpal hand with and without pedicel, length of patella and tibia of leg I) exceeded the correlation threshold of 0.95 in datasets with the body parts and, therefore, three characters (posterior width of carapace, length of palpal hand with pedicel and length of leg I patella) were excluded from further analyses. In CDAs (CDA 1–8), three species mostly formed their own clouds in the ordination space without overlaps (Fig. 8A–H), showing that all the body parts are useful for the differentiation of the three species. The best differentiation of the three species was reached in CDA 6, based on characters measured for leg IV (Fig. 8F), and the weakest differentiation was obtained in CDA 7, based on characters of the tergites (Fig. 8G). For the characters most correlated with the canonical axes and thus contributing to the differentiation of the three species, see Table 2. For details of the correlations of all characters with the axes, see Suppl. material 1. In almost all the classificatory DAs based on the body parts, the percentage of correctly classified specimens reached 100% for all three species. The only exception was the classificatory DA based on characters measured for tergites, for which 80% of specimens were correctly classified into *Lasiochernes cretonatus*, 100% into *Lasiochernes jonicus* and 58.3% into *Lasiochernes pilosus*.

**Table 2.** Results of eight canonical discriminant analyses (CDA 1–CDA 8, Fig. 8) based on morphological characters measured/scored for 19 adult specimens and eight body parts of *Lasiochernes cretonatus*, *Lasiochernes jonicus* and *Lasiochernes pilosus*. The characters which most strongly correlated with the canonical axes (Can 1, Can 2) are listed for each CDA. The extended version of the table, showing all the characters and total canonical structure, is given in Suppl. material 1.

Body parts	Can 1	Can 2
Carapace (CDA 1, Fig. 8A)	Length	Total setae number
	Number of setae on anterior disk	
	Number of setae on posterior margin	
Chelicera (CDA 2, Fig. 8B)	Width	Length of movable finger
	Length/width ratio	
	Number of blades in serrula exterior	
Palp (CDA 3, Fig. 8C)	Length of trochanter	Width of femur
	Length of femur	Length/width ratio of femur
Chela (CDA 4, Fig. 8D)	Length of hand without pedicel	Length/width ratio of hand
	Length of fixed finger	Number of marginal teeth on fixed finger
	Length of chela	Number of antiaxial accessory teeth on movable finger
	Number of antiaxial accessory teeth on fixed finger	
	Number of antiaxial accessory teeth on movable finger	
Leg I (CDA 5, Fig. 8E)	Length of tarsus	Length/depth ratio of femur
Leg IV (CDA 6, Fig. 8F)	Length of trochanter	Length/depth ratio of trochanter
	Depth of trochanter	Length of femur
	Length of tarsus	Depth of tibia
	depth of tarsus	
Tergites (CDA 7, Fig. 8G)	Number of setae on tergite II	Number of setae on tergite III
	Number of setae on tergite V	Number of setae on tergite X
	Number of setae on tergite IX	
Sternites (CDA 8, Fig. 8H)	Number of setae on sternite IV	Lyrifissures number on genital operculum posterior
	Number of setae on sternite X	
	Lyrifissures number on genital operculum posterior	

**Figure 9.**
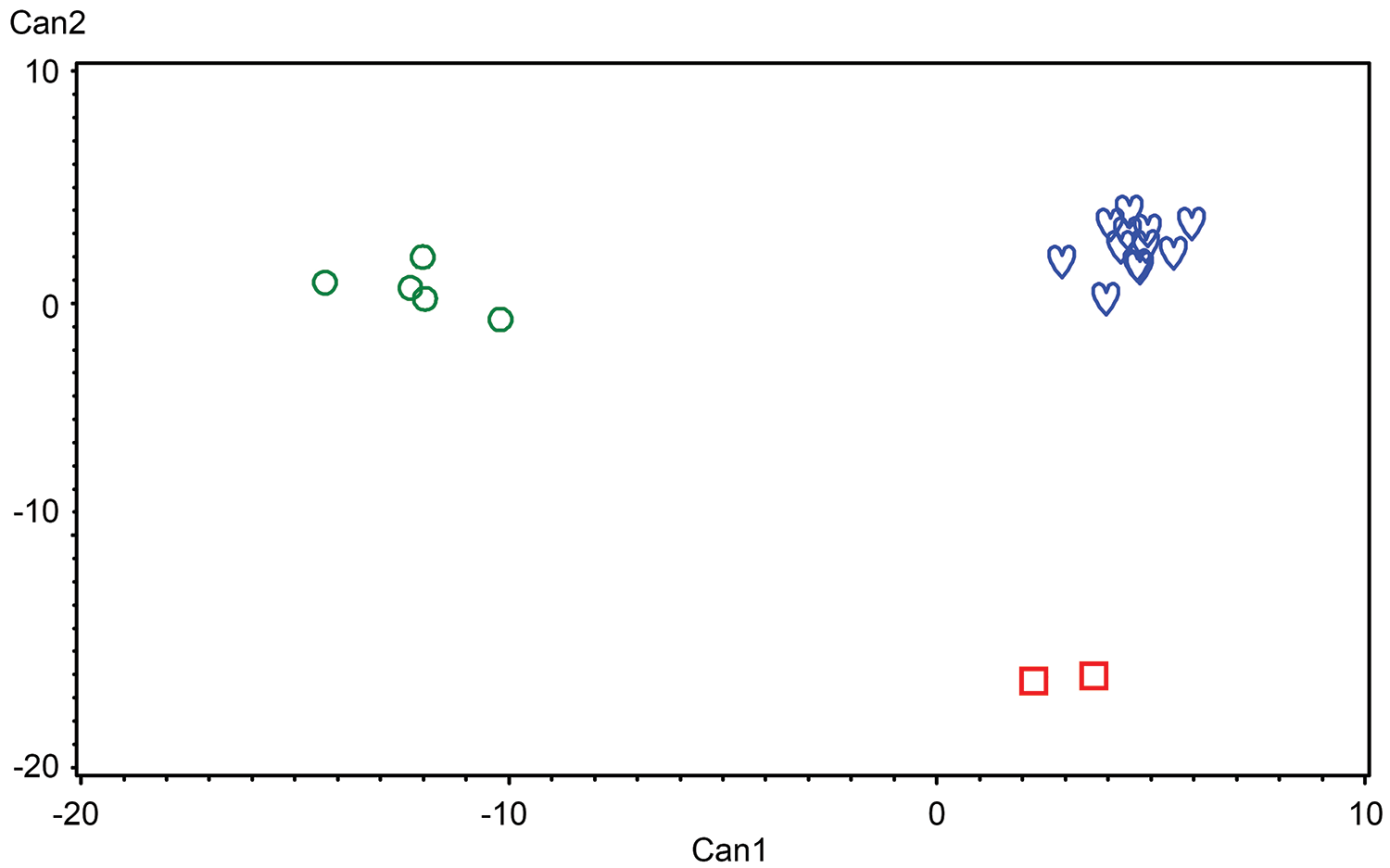
Canonical discriminant analysis (CDA 9) of three *Lasiochernes* species (*Lasiochernes cretonatus*: green circles, *Lasiochernes jonicus*: red squares and *Lasiochernes pilosus*: blue hearts) based on 15 morphological characters for 19 adult specimens. For total canonical structure and the list of characters, see Table 3.

Finally, the classificatory DA 9 and CDA 9 were computed to assess the differentiation of the three species based on the selection of the most important characters from all the parts of the body, as revealed in CDA 1–8. In the classificatory DA 9, the classification success rate reached 100% for all the specimens. The three species were clearly separated in the ordination space of CDA 9 (Fig. 9). The characters most highly correlated with the first and second canonical axis are those in bold type in Table 3.

**Figure 10.**
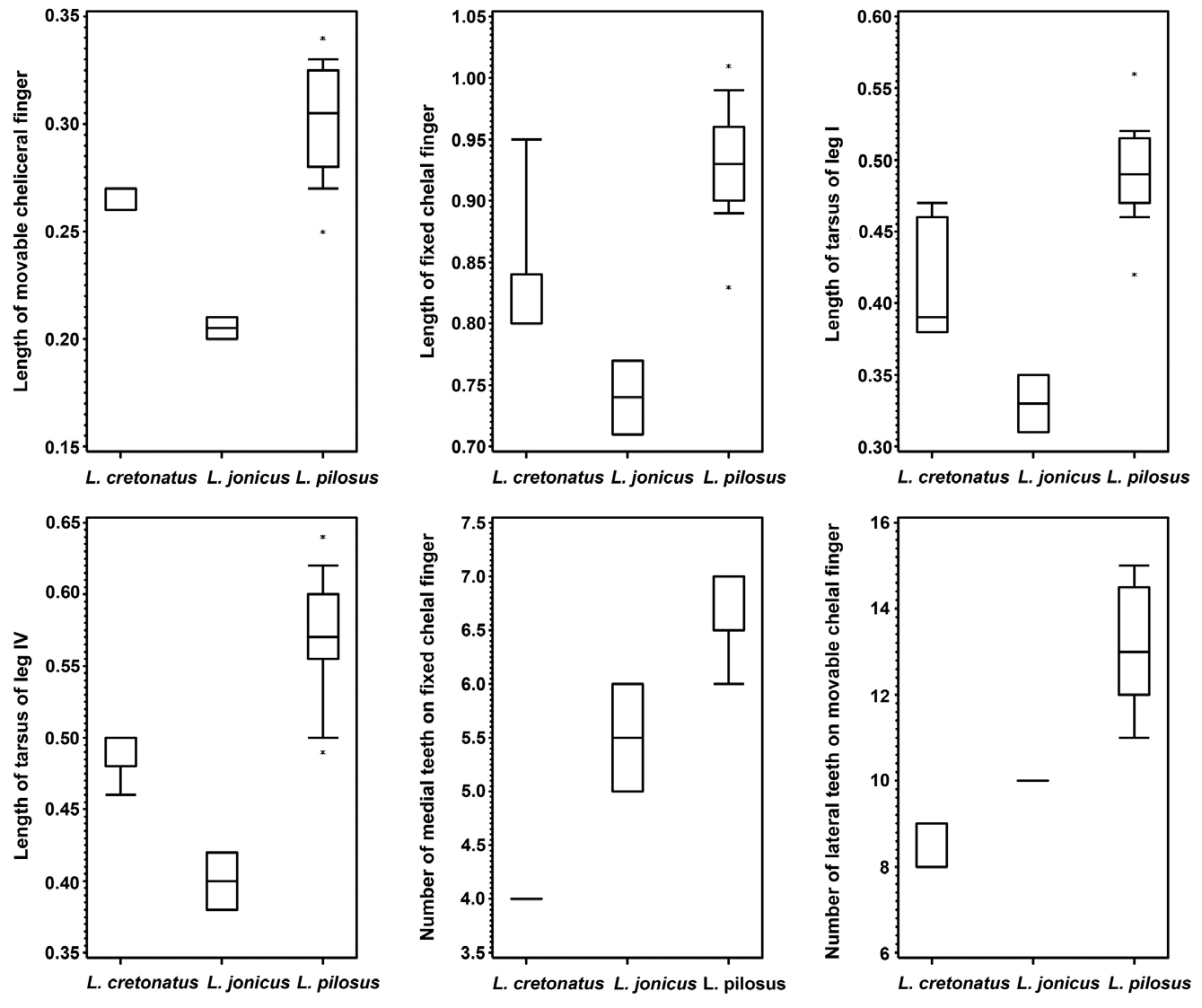
Variation in selected morphological characters of studied *Lasiochernes* species. Rectangles define the 25^th^ and 75^th^ percentiles, horizontal lines show the medians, whiskers are from the 10th to 90th percentiles and asterisks show extreme values (length in mm).

The variations in morphological characters that are most useful for differentiation of the three *Lasiochernes* species are shown in Fig. 10.

**Table 3.** Results of the canonical discriminant analysis (CDA 9, Fig. 9) based on 15 morphological characters measured/scored for 19 specimens of *Lasiochernes cretonatus*, *Lasiochernes jonicus*, and *Lasiochernes pilosus*. Values of the total canonical structure listed in the table express correlations of characters with canonical axes (Can 1, Can 2). Higher total canonical structure values are in bold type.

Morphological characters	Can 1	Can 2
Number of setae on posterior carapace margin	-0.635	-0.420
Total number of setae on carapace	**0.766**	-0.072
Number of setae on anterior disk	**0.829**	0.311
Width of chelicera	0.653	0.523
Length/width ratio of chelicera	-0.649	-**0.641**
Number of blades in serrula exterior	**0.779**	0.484
Length of palpal trochanter	0.661	0.415
Length of palpal femur	0.611	0.388
Length of palpal hand without pedicel	0.625	0.342
Length of palpal chela	0.573	0.500
Number of antiaxial accessory teeth on movable chelal finger	**0.805**	0.355
Depth of tibia of leg I	0.355	**0.584**
Length/depth ratio of femur of leg I	-0.449	0.012
Length of tarsus of leg I	0.411	**0.742**
Length of tarsus of leg IV	0.459	**0.734**

### Identification key to females of *Lasiochernes cretonatus*, *Lasiochernes jonicus*, and *Lasiochernes pilosus*

Based on all the results obtained, nine morphological characters that differentiate females of the three species were selected (Table 4). The values of two of them, namely the length of cheliceral movable finger and the length of the palpal hand with pedicel, do not overlap and therefore allow the unambiguous identification of three *Lasiochernes* females.

**Table:** 

1	Movable finger of chelicera 0.20 mm long; tarsus of leg I 0.35 mm long; femoropatella of leg IV 5.11 times longer than deep	***Lasiochernes jonicus***
‒	Movable finger of chelicera over 0.26 mm long; tarsus of leg I over 0.38 mm long; femoropatella of leg IV less than 4.95 times longer than deep	**2**
2	Palpal hand with pedicel 0.88–0.91 mm long; palpal chela 1.58–1.78 mm long; femur of leg I 1.50–1.65 longer than deep; 71–74 setae on carapace, 31–38 of them situated in front of anterior transverse furrow; tarsus of leg IV with long tactile seta situated one third from base	***Lasiochernes cretonatus***
‒	Palpal hand with pedicel 1.00–1.18 mm long; palpal chela 1.88–2.06 mm long; femur of leg I 1.24–1.46 longer than deep; 81–96 setae on carapace, 49–63 of them situated in front of anterior transverse furrow; tarsus of leg IV with long tactile seta situated approximately in middle of segment	***Lasiochernes pilosus***

**Table 4.** Comparison of adult females of *Lasiochernes cretonatus*, *Lasiochernes jonicus*, and *Lasiochernes pilosus* in values of most differentiating morphological characters (measurements in mm). Boldface values indicate the characters that unambiguously differentiate all the three species.

Characters/species	*Lasiochernes cretonatus*	*Lasiochernes jonicus*	*Lasiochernes pilosus*
Total setae number on carapace	71–74	93	81–96
Number of setae on anterior disk of carapace	31–38	51	49–63
Number of antiaxial accessory teeth on fixed chelal finger	9–13	10	11–16
**Length of movable cheliceral finger**	**0.26–0.27**	**0.20**	**0.28–0.33**
**Length of palpal hand with pedicel**	**0.88–0.91**	**0.99**	**1.00–1.18**
Length of palpal chela	1.58–1.78	1.66	1.88–2.06
Length/depth ratio of femur of leg I	1.50–1.65	1.40	1.24–1.46
Length of tarsus of leg I	0.38–0.47	0.35	0.46–0.56
Length/depth ratio of femoropatella of leg IV	3.76–4.72	5.11	4.20–4.95

## Discussion

### Distribution and habitat preference

*Lasiochernes cretonatus* was described from Souré Cave (Cave of 99 Holy Fathers) in Crete, based on one male collected under a small piece of stone near the cave wall (Henderickx 1998). Šťáhlavský et al. (2005) studied karyotypes of one female and one male tritonymph of *Lasiochernes cretonatus* from the same cave. New specimens were found between organic material, pigeon feathers, dry leaves and pieces of branches in another corner of the same upper cave room, less than six meters from where the holotype was found. Specimens were sifted from leaf litter and collected by vacuuming cracks with a modified portable electric vacuum cleaner.

*Lasiochernes jonicus* was described as Chelifer (Trachychernes) jonicus by Beier (1929) from Agios Mattheos, Corfu, Greece. The types were collected by sifting maquis litter. Later, Beier (1963) specified that, besides the maquis litter, a rotten mouse nest was sifted as well. Altogether 25 males, 19 females and 12 nymphal stages were collected (Beier 1929). Mahnert (1978) recorded three males, one female and 33 nymphs from soil samples in a nameless cave near Profitis Elias church, on Mount Ossa, Thessaly, Greece. The find of our specimens in the Tsouka cave in Pelion, Greece, represents the third known locality of *Lasiochernes jonicus*. The specimens in the Tsouka cave were sifted from material (leaves, small branches and rock fragments between ingrown tree roots) in an upper dry room of the cave.

Ellingsen (1910) described one male of Chelifer (Trachychernes) pilosus from the vicinity of the town of Görz in Austria (now Goricia in Italy) and did not mention the habitat type or the collecting method. Heselhaus (1914) found females and nymphs in mole nests in Netherlands, and described them as *Chelifer falcomontanus*. Later, Berland (1925) recorded several specimens of *Chelifer falcomontanus* from mole nests in Luxembourg and France. Beier (1929) recorded several adults and nymphs in mole nests from Austria and synonymized *Chelifer falcomontanus* with *Chelifer pilosus*. Beier (1929) indicated that the species occurs in mole and ground-squirrel nests. Ressl (1965) and Ressl and Beier (1958) later found many specimens in mole nests with leaf content in Austria. Caporiacco (1949) recorded two *Lasiochernes pilosus* males in the rotten trunk of an oak at Lipizza, Italy (now Lipica, Slovenia). Later Ćurčić (1974) listed *Lasiochernes pilosus* in Slovenia (without providing collecting details) in his catalogue of the former Yugoslavian fauna. There is no mention of this species occurring in Slovenia in the current version of the world pseudoscorpion catalogue (Harvey 2013). The occurrence of *Lasiochernes pilosus* in mole nests in Italy was recorded by Beier (1963) and Inzaghi (1981). In the Netherlands, *Lasiochernes pilosus* was typically collected in mole nests (Van der Hammen 1969). Ventalló (1934) recorded the species for the first time from Spain, based on 14 specimens found in a cave. *Lasiochernes pilosus* also occurs in mole nests in Germany (Hesse 1941, Weidner 1954, Weygoldt 1966, Rehage and Renner 1981). Weygoldt (1966, 1969) reported that the species can be found in water vole nests. *Lasiochernes pilosus* was also collected in Belgium, in mole nests in forests (Cooreman 1946, Leleup 1948, Henderickx 1998, 1999, Šťáhlavský et al. 2005). The locality from which the material studied here was collected is a new record for the geographic distribution of *Lasiochernes pilosus* in Belgium. The locality is located on a hilltop, all specimens were sifted from a mole nest between the roots of a tree on the hilltop, next to a road. Krumpálová and Krumpál (1993) extracted this species from a mole nest for the first time from Slovakia (at Borinka, the same locality as in the current paper). Christophoryová and Krumpál (2010) sifted two females from leaf litter in the Nature Reserve Šúr, Slovakia. New specimens from Borinka were extracted from a mole nest situated in the ecotone between forest and grassland.

### Morphological variation

The original description of *Lasiochernes cretonatus* was based on one male (Henderickx 1998). Comparison of our newly found male with the holotype showed similarity in a majority of the characters (palpal teeth numbers, morphometrics of palps and leg IV, length of body and chelicera and position of tactile seta on tarsus IV). Exceptions were the higher setae number on posterior carapace margin of the newly found male (13 versus 12) and higher number of paraxial accessory teeth on movable chelal finger of the newly found male (4 versus 3). The length of the palpal trochanter was incorrectly given by Henderickx (1998) as 0.84 mm (0.53 mm in the new male). In the present paper, several characters of this species are described for the first time: morphometrics of leg I and carapace, width of chelicera, length of cheliceral finger; number of setae on chelicera, form of galea, rallum, serrula exterior; complete trichobothrial pattern, complete chaetotaxy of carapace, tergites and sternites; numbers of setae and lyrifissures on genital opercula. The female is described here for the first time.

Beier’s (1929, 1963) descriptions of *Lasiochernes jonicus* provided information concerning the cheliceral rallum, serrula exterior, galea, setation and shapes of palpal parts and the position of the tactile setae on tarsus IV and tergite XI. The mean values (for an unspecified number of specimens) of palpal measurements, length of body and carapace of males and females were given by Beier (1929). Mahnert (1978) described one male, giving measurements of the palps and leg IV, and the numbers of marginal and accessory teeth on the chelal fingers. Most of the characters of our male and female correspond with previous descriptions (Beier 1929, 1963, Mahnert 1978); some differences in measurements are probably related to the number of specimens studied. Mahnert (1978) counted more marginal teeth (47 on the fixed finger, versus 42 here, and 50 on the movable finger, versus 47 here), more paraxial accessory teeth (7 on fixed finger, versus 6, and 5 on movable finger, versus 4) and more antiaxial teeth on movable finger (11 versus 10) of the male. In contrast, there are more antiaxial teeth on the fixed finger of our male (12 versus 11). Our results provide information on several new characters: measurements of chelicera, carapace width, measurements of leg I, setae number on carapace, chelicera, tergites, sternites, and genital operculum anterior and posterior.

*Lasiochernes pilosus* was described from one male by Ellingsen (1910), who counted more blades on the serrula exterior than were observed here (27 versus 23–24). Beier (1963) described both sexes, mainly giving their palp measurements. The number of serrula exterior blades was modified to 25–27. The number of antiaxial accessory teeth on the chelal finger was lower than in our specimens (10 versus 11–16 on fixed finger and 8 versus 11–15 on movable finger). The present study provides a number of new details, such as measurements of leg I and IV; the number of paraxial accessory teeth on chelal fingers; the numbers of setae on the carapace, genital opercula, tergites and sternites. For the first time, all nymphal stages are described in detail.

In this paper, the potential of multivariate morphometric techniques for the diagnostic of pseudoscorpion species has been explored. Our study provides a first reference library of morphometric measurements that might be used for the identification of *Lasiochernes* specimens. The PCoA, which depicts the variation without prior definition of the groups in the dataset, showed rather clear differences between the three species. Two large groupings of specimens were visible in the PCoA, the first consisting of *Lasiochernes pilosus* and the second of *Lasiochernes cretonatus* and *Lasiochernes jonicus*. The proximity of the latter two species in PCoA was probably caused by one specimen of *Lasiochernes cretonatus* with significantly higher numbers of setae on the carapace (total and number on anterior disk). Discriminant analyses, which, unlike the PCoA, weight the characters to stress the between-group variation component, revealed considerable differences between the three species. These analyses were also used to identify the most differentiating body parts and the most important characters. The characters traditionally used most in identification keys to pseudoscorpions are those of the palps (Beier 1963, Christophoryová et al. 2011) and their importance was confirmed again by our data. A surprising discovery was that, from among the body parts, the best differentiation of the three species was obtained with leg IV. On the other hand, the tergites were not very useful for species differentiation, due to the high variability of setae number on each tergite. The majority of the most differentiating characters was measured or scored on the carapace, chelicera, chelal fingers and legs I and IV. Until now, the number of setae on the carapace was only rarely used in the descriptions of Chernetidae, mainly the setae number on posterior carapace margin (Beier 1963, Henderickx 1998). The whole count of setae could substantially facilitate the diagnosis of chernetid species in future. The setal counts on the tergites and sternites (except the genital ones) of *Lasiochernes* species showed a high degree of variability.

Multivariate morphometrics have been successfully applied in many other taxonomic studies of various invertebrates. For instance, they were very helpful in interpreting morphological differences between two cryptic species of *Sancassania* Oudemans, 1916, Acari (Klimov et al. 2004). Stekolnikov et al. (2010) revised a species group of chiggers (Acari) using multivariate morphometrics and developed a multivariate classification model to separate three closely related species. These analyses showed complete separation of the studied species. The characters contributing strongly to the discrimination were used in formal description of these species as well as in an identification key. Jagersbacher-Baumann (2014) analyzed four mite species of the *acarorum*-complex (Scutacaridae) using traditional and geometric morphometric methods. The results showed that multivariate morphometric methods are perfectly suitable for differentiating even between morphologically similar scutacarid species, with traditional morphometrics performing better than geometric morphometrics. Van Cann et al. (2015) explored the potential of wing morphometrics for the diagnosis of morphospecies of Tephritidae (Diptera). Multivariate analyses allowed the consistent identification of a significant proportion of specimens in that study. In pseudoscorpion taxonomy, multivariate analyses were used to separate two European Chthoniidae species. Although multivariate analyses suggest specific separation, there was only one unequivocal character for discrimination, the presence or absence of a single isolated tooth on the moveable finger of the chelicerae (Muster et al. 2004).

The genus *Lasiochernes* is noteworthy for its sexual dimorphism (Beier 1963). Males are unambiguously identified by the presence of a long setation arranged on different palpal parts, depending on the species. The setation of the palp is normal in females, without long setae. Our aim was to find characters that could be used for a more reliable identification of the females. It should be noted that our identification key is useful mainly for differentiation of females of *Lasiochernes cretonatus* and *Lasiochernes pilosus*. Values of some characters measured on the female of *Lasiochernes jonicus* are influenced by low number of specimens examined and it is possible that better sampling might show stronger overlaps in future studies. The identification key is based on the characters that were rarely or never used in previously published taxonomic treatments of *Lasiochernes*. Therefore, the comparison of these characters with other European species of the genus is not yet possible.

Based on the results obtained, we assume that future studies will benefit from application of multivariate morphometric analyses, and could potentially help to find new characters and contribute to a more reliable identification of pseudoscorpion species.

## Supplementary Material

XML Treatment for Lasiochernes cretonatus

XML Treatment for Lasiochernes jonicus

XML Treatment for Lasiochernes pilosus

## Supplementary materials

Supplementary material 1 Results of eight canonical discriminant analyses Data type: statistical data Explanation note: Results of eight canonical discriminant analyses (CDA 1–CDA 8, fig. 8) based on morphological characters measured/scored on 19 specimens and eight body segments of *Lasiochernes cretonatus*, *Lasiochernes jonicus*, and *Lasiochernes pilosus*. Values of the total canonical structure listed in the table express correlations of characters with canonical axes (Can 1 and Can 2) in each CDA. Higher total canonical structure values are in bold type. This dataset is made available under the Open Database License (http://opendatacommons.org/licenses/odbl/1.0/). The Open Database License (ODbL) is a license agreement intended to allow users to freely share, modify, and use this Dataset while maintaining this same freedom for others, provided that the original source and author(s) are credited.Jana Christophoryová, Katarína Krajčovičová, Hans Henderickx, Stanislav Španiel
