# Supplementary material for: A multivariate study of differentiating characters between three European species of the genus Lasiochernes Beier, 1932 (Pseudoscorpiones, Chernetidae)
Source: Zookeys. 2016 Nov 7;(629):51–81. doi: 10.3897/zookeys.629.8445 (PMC5126538; doi:10.3897/zookeys.629.8445)
Supplement: Supplementary material 1 — Results of eight canonical discriminant analyses [file zookeys-629-051-s001.docx]

Supplementary file 1. Results of eight canonical discriminant analyses (CDA 1–CDA 8, fig. 8) based on morphological characters measured/scored on 19 specimens and eight body segments of *Lasiochernes cretonatus*, *L. jonicus* and *L. pilosus*. Values of the total canonical structure listed in the table express correlations of characters with canonical axes (Can 1 and Can 2) in each CDA. Higher total canonical structure values are in bold type.

| **Morphological characters** | **Can 1** | **Can 2** |
| --- | --- | --- |
| **Carapace** (CDA 1, fig. 8A) |  |  |
| Length | **0.877** | -0.205 |
| Length/posterior width ratio | -0.400 | 0.651 |
| Total setae number | 0.549 | **-0.811** |
| Setae number in front of anterior transverse furrow | **0.864** | -0.464 |
| Setae number on posterior carapace margin | **-0.789** | 0.161 |
|  |  |  |
| **Chelicera** (CDA 2, fig. 8B) |  |  |
| Length | 0.320 | -0.078 |
| Width | **0.850** | 0.045 |
| Length/width ratio | **-0.917** | -0.161 |
| Length of movable finger | 0.728 | **0.500** |
| Number of terminal rami on galea | 0.771 | -0.059 |
| Number of blades on serrula exterior | **0.930** | -0.081 |
|  |  |  |
| **Palp** (CDA 3, fig. 8C) |  |  |
| Length of trochanter | **0.802** | 0.028 |
| Width of trochanter | 0.495 | 0.272 |
| Length/width ratio of trochanter | 0.654 | -0.204 |
| Length of femur | **0.744** | 0.022 |
| Width of femur | 0.259 | **0.650** |
| Length/width ratio of femur | 0.290 | **-0.629** |
| Length of patella | 0.397 | 0.197 |
| Width of patella | 0.520 | -0.046 |
| Length/width ratio of patella | -0.391 | 0.356 |
|  |  |  |
| **Chela** (CDA 4, fig. 8D) |  |  |
| Length of hand without pedicel | **0.701** | -0.223 |
| Width of hand | 0.560 | -0.155 |
| Length/width ratio of hand with pedicel | 0.536 | **-0.324** |
| Length of fixed finger | **0.788** | 0.265 |
| Length of chela | **0.773** | -0.043 |
| Length of chela/width hand ratio | 0.530 | 0.154 |
| Number of marginal teeth on fixed finger | 0.117 | **0.366** |
| Number of lateral accessory teeth on fixed finger | **0.728** | -0.259 |
| Number of marginal teeth on movable finger | -0.555 | 0.214 |
| Number of lateral accessory teeth on movable finger | **0.842** | **-0.362** |
|  |  |  |
| **Leg I** (CDA 5, fig. 8E) |  |  |
| Length of trochanter | 0.656 | 0.320 |
| Width of trochanter | 0.628 | 0.530 |
| Length/width ratio of trochanter | 0.171 | -0.365 |
| Length of femur I | 0.591 | 0.257 |
| Width of femur I | 0.597 | 0.618 |
| Length/width ratio of femur I | -0.221 | **-0.784** |
| Width of femur II | 0.656 | 0.367 |
| Length/width ratio of femur II | -0.419 | -0.390 |
| Length of tibia | 0.540 | 0.046 |
| Width of tibia | 0.682 | 0.134 |
| Length/width ratio of tibia | -0.332 | -0.137 |
| Length of tarsus | **0.853** | 0.131 |
| Width of tarsus | 0.614 | -0.249 |
| Length/width ratio of tarsus | 0.470 | 0.354 |
|  |  |  |
| **Leg IV** (CDA 6, fig. 8F) |  |  |
| Length of trochanter | **0.533** | 0.322 |
| Width of trochanter | **0.512** | 0.577 |
| Length/width ratio of trochanter | -0.125 | **-0.647** |
| Length of femur | 0.204 | **0.799** |
| Width of femur | 0.382 | 0.576 |
| Length/width ratio of femur | -0.381 | 0.341 |
| Length of tibia | 0.429 | 0.427 |
| Width of tibia | 0.131 | **0.648** |
| Length/width ratio of tibia | 0.360 | -0.425 |
| Length of tarsus | **0.691** | 0.524 |
| Width of tarsus | **0.522** | 0.295 |
| Length/width ratio of tarsus | 0.348 | 0.396 |
|  |  |  |
| **Tergites** (CDA 7, fig. 8G) |  |  |
| Setae number on tergite I | 0.067 | 0.321 |
| Setae number on tergite II | **0.678** | -0.264 |
| Setae number on tergite III | 0.126 | **-0.390** |
| Setae number on tergite IV | 0.517 | -0.091 |
| Setae number on tergite V | **0.684** | -0.083 |
| Setae number on tergite VI | 0.608 | -0.116 |
| Setae number on tergite VII | 0.483 | -0.233 |
| Setae number on tergite VIII | 0.553 | 0.147 |
| Setae number on tergite IX | **0.837** | 0.201 |
| Setae number on tergite X | 0.511 | **0.400** |
|  |  |  |
| **Sternites** (CDA 8, fig. 8H) |  |  |
| Setae number on sternite IV | **-0.420** | 0.133 |
| Setae number on sternite V | -0.198 | 0.335 |
| Setae number on sternite VI | -0.131 | 0.105 |
| Setae number on sternite VII | -0.218 | 0.222 |
| Setae number on sternite VIII | -0.045 | 0.258 |
| Setae number on sternite IX | 0.150 | 0.023 |
| Setae number on sternite X | **0.421** | -0.269 |
| Setae number on sternite XI | 0.354 | -0.252 |
| Setae number on genital operculum anterior | -0.313 | 0.033 |
| Setae number on genital operculum posterior | -0.256 | 0.324 |
| Lyrifissures number on genital operculum anterior | 0.367 | -0.156 |
| Lyrifissures number on genital operculum posterior | **0.409** | **0.460** |
